# Expanding the phenotypic spectrum associated with *ZIC1* variants: A neurodevelopmental disorder with and without craniosynostosis

**DOI:** 10.1016/j.gim.2026.102585

**Published:** 2026-06

**Authors:** Laura M. Watts, Michelle S.M. Chang, Elizabeth Lewis-Orr, Isaac S. Walton, Lisa Leinhos, Rebecca S. Tooze, Yang Pei, Eduardo Calpena, J. Heather Vedovato-dos-Santos, Dora Steel, Kimberley M. Reid, Manju A. Kurian, Shekeeb S. Mohammad, Vincent Cantagrel, Karine Siquier, Nathalie Boddaert, Marlene Rio, Moira Blyth, Alison Kraus, Fuad Al Mutairi, Susan E. Holder, Virginia E. Clowes, Jan M. Cobben, Andrew T. Timberlake, Ellen R. Elias, Helen Stewart, Diana Johnson, Julie S. Cohen, Kristin W. Barañano, Sophia Ceulemans, Marilyn C. Jones, Rita I. Ortega Rico, Marte G. Haug, Siren Berland, Hannah M. Bombei, Anna Paulson, Alpa Sidhu, Catherine F. Gooch, Kátia M. da Rocha, Maria Rita Passos Bueno, Alexandra Ţopa, Aida Z. Muslimovic, Giovanni Maltese, Tiong Yang Tan, Emma McCann, Helen Lord, Hui-lin Chin, Jeremy Lin, Denise Li-Meng Goh, Boris Keren, Perrine Charles, Trayan Delchev, Daniela Avdjieva-Tzavella, Salem Alawbathani, Ligia Almeida, Ameni Kdissa, Ruslan Al-Ali, Aida M. Bertoli-Avella, David Johnson, Andrew O.M. Wilkie, Ruth M. Arkell, Deborah J. Shears, Stephen R.F. Twigg

**Affiliations:** 1 Oxford Centre for Genomic Medicine, Oxford University Hospitals NHS Foundation Trust, Oxford, United Kingdom; 2 John Curtin School of Medical Research, Garran Rd, The Australian National University, Acton, ACT, Australia; 3 Clinical Genetics Group, MRC Weatherall Institute of Molecular Medicine, University of Oxford, Oxford, United Kingdom; 4 Grupo de Investigación en Biomedicina Molecular, Celular y Genómica, Unidad CIBERER, Instituto de Investigación Sanitaria La Fe (IIS La Fe), Valencia, Spain; 5 Molecular Neurosciences, Developmental Neurosciences, Zayed Centre for Research into Rare Disease in Children, UCL Great Ormond Street Institute of Child Health, London, United Kingdom; 6 Department of Neurology, Great Ormond Street Hospital, London, United Kingdom; 7 Kids Neuroscience Centre, The Children’s Hospital at Westmead, Faculty of Medicine and Health, University of Sydney, Sydney, NSW, Australia; 8 Université Paris Cité, INSERM UMR1163, Institut Imagine, Developmental Brain Disorders Laboratory, Paris, France; 9 Département de Radiologie Pédiatrique, INSERM UMR 1163 and INSERM U1299, Institut Imagine, AP-HP, Necker Enfant Malade Hospital, Paris, France; 10 Service de Médecine Génomique des Maladies Rares, Fédération de Génétique et Médecine Génomique, AP-HP, Hôpital Necker Enfants Malades, Paris, France; 11 North of Scotland Regional Genetics Service, Ashgrove House, Foresterhill, Aberdeen, United Kingdom; 12 Yorkshire Regional Genetics Service, Chapel Allerton Hospital, Leeds, United Kingdom; Castle Hill Hospital, Cottingham, Hull, United Kingdom; 13 Genetics and Precision Medicine Department of Pediatrics, King Abdullah Specialized Children Hospital, King Abdulaziz Medical City, MNGHA, Riyadh, Saudi Arabia; 14 King Abdullah International Medical Research Center (KAIMRC), King Saud bin Abdulaziz University for Health Sciences, MNGHA, Riyadh, Saudi Arabia; 15 North West Thames Regional Genetics Service, London North West Healthcare NHS Trust, London, United Kingdom; 16 Section of Genetics and Genomics, Department of Metabolism Digestion and Reproduction, Faculty of Medicine, Imperial College, London, United Kingdom; 17 Hansjörg Wyss Department of Plastic Surgery, NYU Langone Medical Center, New York, NY; 18 Departments of Pediatrics and Genetics, University of Colorado School of Medicine, Aurora, CO; 19 Sheffield Clinical Genetics Service, Sheffield Children's NHS Foundation Trust, Sheffield, United Kingdom; 20 Department of Neurology and Developmental Medicine, Kennedy Krieger Institute, Baltimore, MD; 21 Department of Neurology, Johns Hopkins University School of Medicine, Baltimore, MD; 22 Division of Genetics at Rady Children's Hospital, San Diego, CA; 23 Department of Pediatrics at University of California, San Diego, CA; 24 Fundación Centro Colombiano de Epilepsia y Enfermedades Neurológicas, FIRE, Cartagena de Indias, Colombia; 25 Department of Medical Genetics, St. Olav's University Hospital, Trondheim, Norway; 26 Department of Medical Genetics, Haukeland University Hospital, Bergen, Norway; 27 Division of Medical Genetics and Genomics, Stead Family Department of Pediatrics, University of Iowa Healthcare, Iowa City, IA; 28 Division of Genetics and Genomic Medicine, Department of Pediatrics, Washington University in St Louis, St Louis, MO; 29 Human Genome and Stem Cell Research Center, University of São Paulo, Institute of Bioscience, Department of Genetics and Evolutionary Biology, São Paulo, Brazil; 30 Department of Laboratory Medicine, University of Gothenburg, Sahlgrenska Academy, Gothenburg, Sweden; 31 Department of Clinical Genetics and Genomics, Sahlgrenska University Hospital, Gothenburg, Sweden; 32 Department of Plastic Surgery, University of Gothenburg, Sahlgrenska Academy, Gothenburg, Sweden; 33 Victorian Clinical Genetics Services, Murdoch Children’s Research Institute, Royal Children’s Hospital, Melbourne, Australia; 34 Department of Paediatrics, University of Melbourne, Melbourne, Australia; 35 Department of Clinical Genetics, Liverpool Women's NHS Foundation Trust, Liverpool, United Kingdom; 36 Oxford Genetics Laboratories, Oxford University Hospitals NHS Foundation Trust, The Churchill Hospital, Oxford, United Kingdom; 37 Division of Genetics and Metabolism, Khoo Teck Puat-National University Children’s Medical Institute, National University Hospital, Singapore; 38 Department of Paediatrics, Yong Loo Lin School of Medicine, National University of Singapore, Singapore; 39 Assistance Publique-Hôpitaux de Paris, Département de Génétique, Hôpital Pitié-Salpêtrière, Paris, France; 40 Department of Clinical Genetics, University Pediatric Hospital, Sofia, Bulgaria; 41 CENTOGENE GmbH, Rostock, Germany; 42 Craniofacial Unit, John Radcliffe Hospital, Oxford University Hospitals NHS Foundation Trust, Oxford, United Kingdom; 43 NIHR Oxford Biomedical Centre, Oxford, United Kingdom

**Keywords:** Craniosynostosis, gain-of-function, loss-of-function, neurodevelopmental disorder, ZIC1

## Abstract

**Purpose:**

*ZIC1* encodes a transcription factor with critical roles in vertebrate neural and skeletal development. Heterozygous deletions encompassing *ZIC1* and *ZIC4* cause Dandy-Walker malformation, whilst in the final exon heterozygous *ZIC1* variants result in a distinct phenotype of craniosynostosis with variable intellectual disability via a gain-of-function mechanism. We describe the largest group of individuals harboring *ZIC1* variants to date, significantly expanding the phenotypic spectrum and allowing genotype-phenotype correlation.

**Methods:**

Through international collaboration we identified 18 different heterozygous *ZIC1* variants from 22 families, comprising 30 individuals.

**Results:**

Twelve families segregated a phenotype comprising craniosynostosis with facial dysmorphism, structural brain abnormalities and developmental delay, whereas 10 families had a neurodevelopmental disorder alone without craniosynostosis. Variants associated with craniosynostosis were clustered in the final exon (3) and were predominantly truncating variants predicted to escape nonsense-mediated decay. Variants associated with neurodevelopmental disorder alone included missense substitutions within exons 1 and 2 predicted to disrupt the normal function of the zinc-finger domain, leading to loss of ZIC1 function, which was confirmed in a functional assay.

**Conclusion:**

This study presents evidence for a *ZIC1* genotype-phenotype correlation differentiating variants that cause a neurodevelopmental phenotype with and without craniosynostosis.

## Introduction

*ZIC1* (HGNC:12872) encodes a member of the zinc finger (ZF) of cerebellum (ZIC) family of transcription factors with critical roles in vertebrate neural and skeletal development.^1^, ^2^, ^3^, ^4^ In the human genome, *ZIC* genes are arranged as 2 pairs (*ZIC1/4* (HGNC:20393) and *ZIC2/5* (HGNC:12873/20322) and an unpaired *ZIC3* (HGNC:12874); the corresponding murine orthologs (*Zic1*-*Zic5*) have similar positional relationships. All five ZIC proteins share a highly conserved ZF DNA-binding domain, which functions in protein interaction,^5^ nuclear localization,^6^ and DNA binding.^3^ Heterozygous disruption of ZIC1-3 protein function has serious consequences for human health, causing a range of congenital anomalies including brain malformations, holoprosencephaly (OMIM 609637), and left-right patterning defects (OMIM 306955).^2^^,^^4^

Heterozygous deletions of 3q25.1, encompassing both *ZIC1* and *ZIC4*, result in cerebellar abnormalities including Dandy-Walker malformation (DWM, OMIM 220200),^4^ whereas heterozygous intragenic *ZIC1* variants that are predominantly in the third (last) exon and truncating, cause a distinct phenotype of craniosynostosis with variable intellectual disability (ID), likely to occur through a gain-of-function mechanism (OMIM 616602, 618736).^7^ The key features of this syndrome are craniosynostosis (most commonly affecting both coronal sutures), dysmorphic facial features, structural brain malformations affecting the corpus callosum, ventricles and posterior fossa, and variable ID.^7^, ^8^, ^9^, ^10^, ^11^, ^12^ Additional reported features include strabismus, scoliosis, and a distinctive phenotype of calvarial ossification defects ranging from widened fontanelles with delayed closure to enlarged parietal foramina and caput membranaceum. Although heterozygous *ZIC1* variants have most commonly been reported as occurring de novo, there have been individual reports of familial inheritance with variable expressivity, and mosaicism, but the frequency with which this occurs, with important implications for reproductive counseling, is unclear.^7^, ^8^, ^9^, ^10^, ^11^, ^12^ Importantly, it has been demonstrated in 2 cases that *ZIC1* nonsense variants in the final exon escape from nonsense-mediated decay (NMD),^7^^,^^9^ predicted to result in production of truncated/mutant protein and a phenotype distinct from heterozygous deletion of *ZIC1*. Support for such a mechanism for the craniosynostosis-associated variants was obtained from assays of Zic1 function in Xenopus, which showed increased expression of the homeodomain transcription factor *En-2* after injection of mutant constructs.^7^

Consistent with human 3q25.1 deletions causing cerebellar malformations, mice that are homozygous *Zic1* knockouts or trans-heterozygous for loss of *Zic1* and *Zic4* demonstrate cerebellar hypoplasia from reduced cellular proliferation together with cerebellar foliation abnormalities.^1^^,^^4^ Because there are no skull abnormalities in these mice, the occurrence of pathogenic variants of *ZIC1* in craniosynostosis was surprising and revealed a previously unappreciated role for ZIC1 in cranial suture morphogenesis. Murine *Zic1* is expressed within the meningeal layers at embryonic (E) day 12.5 at the forming coronal suture^13^ and has been detected in human embryonic suture mesenchyme.^14^ At later embryonic stages, *Zic1* expression has been detected in osteoprogenitor cells and within the ligament-like and meningeal cell populations above and below the suture, respectively.^15^ The role of Zic1 in coronal suture biogenesis is not clear; expression overlaps with *Engrailed 1* (*En1*),^7^ a key molecule in the establishment of the coronal suture,^4^ which is known to be induced by *Zic1* in Drosophila^16^ and Xenopus.^2^^,^^4^ However, coexpression with *Lmx1b* may also indicate a role as an antiosteogenic factor.^13^

Since the original description of pathogenic heterozygous *ZIC1* variants and craniosynostosis in 5 families,^7^ there have been a further 10 affected individuals reported with a combination of craniosynostosis, structural brain malformations, and ID or neurodevelopmental disorder (NDD).^8^, ^9^, ^10^, ^11^, ^12^^,^^16^, ^17^, ^18^ Here, were present a further 22 families, comprising 30 affected individuals, significantly expanding the phenotypic spectrum. Moreover, we demonstrate a genotype-phenotype correlation and show that proximal loss-of-function variants in *ZIC1* result in a novel phenotype of NDD without craniosynostosis.

## Materials and Methods

### Participants

Participants were identified through contact with the Oxford Clinical Genetics Group or through GeneMatcher (https://genematcher.org/) and ClinVar (https://www.ncbi.nlm.nih.gov/clinvar/). All participants consented to inclusion in this study. None has previously been reported in detail in the medical literature (2 were identified in large sequencing studies^16^^,^^18^), and all are unrelated to earlier reported cases.^7^, ^8^, ^9^, ^10^, ^11^, ^12^ Clinical studies were approved by London-Riverside REC (09/H0706/20, Genetic Basis of Craniofacial Malformation), and East of England-Cambridge South REC (14/EE/1112, 100kGP). Written informed consent was obtained for each participating individual. Permission was obtained to publish participant photos or images.

DNA from Families 2 and 20 was analyzed by polymerase chain reaction and deep sequencing to investigate segregation (methodology included within Supplemental Figure 1).

### Variant classification

**Figure 1:**
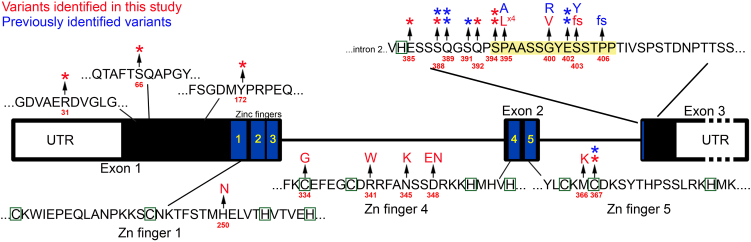
**Novel variants identified in *ZIC1*.** A. *ZIC1* comprises 3 exons (coding regions are highlighted in black) and encodes a Zn finger transcription factor (the 5 Zn finger domains are shown as blue boxes). The variants identified in this study and previously are indicated in red and blue, respectively, in the amino acid sequences shown above and below the gene structure. The canonical cysteines and histidine residues that define the 1st, 4th, and 5th C2H2 Zn finger motifs are boxed. The Subclass A C-terminal Conserved domain^18^ in exon 3 is highlighted in yellow. Variants found in participants with craniosynostosis primarily cluster between residues 385-400 in the last exon (top), although 2 new variants in individuals with metopic synostosis were identified in Zn finger 5, including 1 identical to a previously published variant p.(Cys367Ter).^8^ In accordance with published data,^7^, ^8^, ^9^, ^10^, ^11^, ^12^^,^^16^^,^^17^^,^^22^ craniosynostosis-associated variants are mainly, but not exclusively, truncations. A combination of missense and truncating variants across all exons is found in individuals with NDD without craniosynostosis.

Variants were classified using the Association for Clinical Genomic Science Best Practice Guidelines for Variant Classification in Rare Disease, which provide additional guidance for interpretation of the 2015 American College of Medical Genetics criteria.^19^^,^^20^ PVS1 was applied to exon 1 loss-of-function variants,^21^ with reduced strength incorporating previous evidence of loss of function (from 3q25 deletions and mouse models), whether the transcript was predicted to undergo NMD and the domains and proportion of protein affected. Nonsense and truncating variants in the final exon were interpreted according to Figure 1 of the Association for Clinical Genomic Science guidance. Specifically, PM4 was applied for final exon nonsense variants based upon previous evidence of gain-of-function. Further information on classification, a summary of the evidence and criteria applied in classification is found in Supplemental Table 1.

### Functional studies

Luciferase assays were performed as previously described^23^ with slight modifications. HEK293T cells grown in 12-well cell culture plates (Costar, CLS3513) were transfected with a total of 1.6 μg of DNA per well: 0.8 μg of the reporter construct and 0.8 μg of the expression construct. 6 hours after transfection, cells were dissociated from the growth surface using 0.05 g/L trypsin, and each transfection was plated in triplicate onto a flat bottom tissue culture-treated 96-well plate (Costar, CLS3628). Luminescence was measured 24 hours after transfection in each of the 3 replicate samples from 3 separate transfections by lysing the cells with 100 μL of a 1:1 dilution of DMEM and luciferase substrate (ONE-Glo Luciferase Assay System, Promega). Luminescence was measured using a TECAN Infinite M1000 Pro plate reader. Zic1-C339S was used as a control for loss of binding; Cys339 is a key cysteine residue in ZF 4, substitution of which eliminates the DNA binding ability of both ZIC1 and ZIC2 (C370S).^23^ Expression of ZIC1 protein in nuclear fractions was analyzed by Western blot as previously described,^23^ using α-V5 (1:3000 dilution; Life Technologies, R960-25); α-TATA binding protein (1:2000 dilution; Abcam, ab818) was used to assess sample loading amounts. Raw data, statistical analysis, representative Western blots, and cellular localization studies are shown in Supplemental Figure 2.

To assess the effect of the missense substitutions in the ZFs of ZIC1, in silico structural analysis was performed using MichalaNGLo-Venus (https://michelanglo.sgc.ox.ac.uk/)^24^ and Protein Databank 3D viewer (https://www.rcsb.org/3d-view), based on the ZIC3 ZF 1-4 structure (https://www.rcsb.org/structure/2RPC)^6^ or the alphaFold structural prediction for ZIC1 (https://alphafold.ebi.ac.uk/entry/Q15915). Assessment of DNA binding specificity was performed using a web tool that calculates position weight matrices (http://zf.princeton.edu).^25^

## Results

### Variants in *ZIC1*

Eighteen different heterozygous *ZIC1* variants were identified in 30 individuals from 22 families comprising 8 nonsense, 9 missense, and 1 frameshift alteration (Figure 1,^18^ Table 1^16^). In total, 17 of 18 identified variants are considered pathogenic or likely pathogenic. In comparison with previously published variants that primarily locate within exon 3 (Figure 1,^7^, ^8^, ^9^, ^10^, ^11^, ^12^^,^^16^, ^17^, ^18^, ^22^^,^^22^ Supplemental Table 2), the 14 novel variants we describe are spread throughout the gene, including 3 nonsense variants within exon 1, and 7 missense variants in ZF domains encoded by exons 1 and 2. Twelve of the novel variants arose de novo (Table 1). Clinical features are described in Table 2 and Supplemental Table 3.

**Table 1:** Heterozygous *ZIC1* variants identified in this study

Family #	Individuals With Variants († = Index Patient)	Exon	HGVS (NC_000003.12) GRCh38	cDNA Change (NM_003412.4)	Amino Acid Change (NP_003403.2)	CADD Score	Alpha Missense	gnomAD Prevalence (v4.1.0)	De Novo?	Phenotype^a^		Classification^c^
										NDD	CRS	
**1**	II-1†	1	g.147410203C>T	c.91C>T	p.(Arg31Ter)	35	-	0		Y	N	LP (PM2, PVS1 (strong))
**2**	I-1, II-1†, II-2†	1	g.147410309C>A	c.197C>A	p.(Ser66Ter)	36	-	0	N	Y	N	LP (PM2, PVS1 (strong))
**3**	II-1, III-1†	1	g.147410628C>G	c.516C>G	p.(Tyr172Ter)	36	-	0	Y (in II-1)	Y	N	LP (PM2, PVS1 (strong), PM6)
**4**	II-1†	1	g.147410860C>A	c.748C>A	p.(His250Asn)	26.8	0.915	0	Y	Y	N	LP (PM2, PP3, PS3, PM6)
**5**	II-1†	2	g.147412535T>G	c.1000T>G	p.(Cys334Gly)	32	0.998	0	Y	Y	N	LP (PM2, PP3, PM1 (strong), PM6)
**6**	II-1†	2	g.147412556C>T	c.1021C>T	p.(Arg341Trp)	31	0.993	0		Y	N	LP (PM2, PP3, PS3)
**7**	II-1†	2	g.147412570C>G	c.1035C>G	p.(Asn345Lys)	24	0.999	0	Y	Y	N	LP (PM2, PP3, PM1, PM6)
**8**	II-1†	2	g.147412577G>A	c.1042G>A	p.(Asp348Asn)	32	0.995	0	Y	Y	Y	P (PM2, PP3, PS3, PS2)
**9**	II-1†	2	g.147412579C>G	c.1044C>G	p.(Asp348Glu)	23	0.992	0	Y	Y	N	LP (PM2, PP3, PS3, PM6)
**10**	II-1†	2	g.147412632T>A	c.1097T>A	p.(Met366Lys)	25.4	0.6051	0.000001239		Y	Y	VUS (PP3, PS3)
**11**	II-1†	2	g.147412636C>A	c.1101C>A	p.(Cys367Ter)	37	-	0	Y	Y	Y	LP (PM2, PS4 (moderate), PP3, PM6)
**12**	I-2, II-2†	3	g.147413360G>T	c.1153G>T	p.(Glu385Ter)	25.2	-	0	Y (in I-2)^c^	Y	Y	LP (PM2, PM4, PP3, PS2)
**13**	II-1†, II -2	3	g.147413370C>A	c.1163C>A	p.(Ser388Ter)	31	-	0		Y	Y	LP (PM2, PS4 (supporting), PM4, PP3, PM6)
**14**	II-1†	3	g.147413381C>T	c.1174C>T	p.(Gln392Ter)	45	-	0	Y	Y	Y	LP (PM2, PM4, PP3, PM6)
**15**	II-1†	3	g.147413388C>A	c.1181C>A	p.(Ser394Ter)	39	-	0		unknown	Y	LP (PM2, PS4 (supporting), PM4, PP3, PP4)
**16**	II-1†	3	g.147413388C>A	c.1181C>A	p.(Ser394Ter)	39	-	0		Y	Y	
**17**	II-1†	3	g.147413391C>T	c.1184C>T	p.(Pro395Leu)	29.5	0.197	0		Y	Y	LP (PM2, PS4 (moderate), PM5 (supporting), PP2, PM6)
**18**	II-1†	3	g.147413391C>T	c.1184C>T	p.(Pro395Leu)	29.5	0.197	0	Y	Y	Y	
**19**	I-2, II-1†, II-2	3	g.147413391C>T	c.1184C>T	p.(Pro395Leu)	29.5	0.197	0		Y	N^b^	
**20**	I-2, II-1†	3	g.147413391C>T	c.1184C>T	p.(Pro395Leu)	29.5	0.197	0	Y (in I-2)	Y	Y	
**21**	II-1†	3	g.147413406G>T	c.1199G>T	p.(Gly400Val)	29.2	0.466	0		Y	Y	LP (PM2, PM5, PP2,PP4)
**22**	II-1†	3	g.147413414_147413415insA	c.1207_1208insA	p.(Ser403TyrfsTer41)	-	-	0	Y	Y	N^b^	LP (PM2, PVS1 (moderate), PM6)

*LP*, likely pathogenic; *P*, pathogenic; *VUS*, variant of uncertain significance: Supplemental Table 1 for details.

a NDD, neurodevelopmental disorder - at least one member in a family with one or more impairments in cognition, behavior, communication, and motor function; CRS, craniosynostosis.

b Plagiocephaly or brachycephaly noted.

c The mother was mosaic for the pathogenic variant. This family was published previously,^16^ but further clinical detail are included here.

**Table 2:** Summary of clinical features

Family #	Individual	Sex	Age at Assessment	Skull Shape	Sutures Fused	Dysmorphism	Structural Brain Abnormality	Global Developmental Delay/ID^a^	ASD/ ADHD	Ocular Features	Tone	Other MSK Features
**1**	II-1	M	7y	Normal	NA	N	Y	++	ASD	N	↓	N
**2**	I-1^b^	M	NA	NK	NK	NK	NK	NK	NK	NK	NK	NK
	II-1	M	11y 10m	Normal	NA	N	Y	+/−	N	N	Normal	N
	II-2	M	14y 3m	Normal	NA	N	Y	N	N	N	Normal	N
**3**	II-1	M	adult	Normal	NA	N	NK	N	N	N	Normal	N
	III-1	M	5y	Plagiocephaly	NA	Y	Nonspecific MRI changes	+++	ASD	RE	NK	N
**4**	II-1^c^	F	7y, deceased 8y	Narrow bitemporal diameter	NA	Y	Y	+++	N	Sb, CB	↑	Sc, rocker bottom feet
**5**	II-1	M	20y	Mild brachycephaly	NA	Y	Y	++	ADHD	Sb	↓	RM, BD HM
**6**	II-1	M	6y 8m	Normal	NA	Y	NK	+	ASD	NK	↓	HM
**7**	II-1	M	5y 4m	Macrocephaly	NA	N	Y	+/−	ADHD	ON	↓	Tc, VA
**8**	II-1	M	12y	Brachycephaly	S, BC	Y	Y	+++	ASD	Sb	↑	Sc, HM (hip)
**9**	II-1	M	7y 7m and 18y	Metopic ridge, closed AF at birth	NA	Y	Y	++	ASD, ADHD	Sb, RE^g^	↓	HM, Tl
**10**	II-1	M	4m, deceased 2y	Metopic ridge, trigonocephaly	Me	Y	Y	++	NA	ON^f^	↑	N
**11**	II-1		19m	Trigonocephaly	Me	Y	Y	++	ASD	Sb	↑	N
**12**	I-2^d^	F		Normal	NA	N	NK	N	N	N	Normal	N
	II-2	M	3y 7m	Oxycephaly, plagiocephaly	BC, RL	Y	Y	SD	N	Sb, ON^f^	Normal	Sc, VA
**13**	II-1	M	13m and 5y 9m	Brachycephaly	BC	Y	Y	SD	N	Sb	Normal	Cl
	II-2	M	13m and 5y 9m	Brachycephaly	BC	y	Y	SD	N	Sb, RE	Normal	Resolved Sc, Cl
**14**	II-1	F	6m	Turribrachycephaly	BC, LL	Y	Y	MD	N	Sb,ON, RE^f^	Normal	Mild Sc
**15**	II-1^e^		1m	NK								
**16**	II-1	F	8y 5m	Brachyturricephaly	PS	Y	Y	+	Suspected ASD	ON, Sb	Normal	NK
**17**	II-1	M	First assessed 3m, follow up to 9y	Brachycephaly, plagiocephaly	LC, Me	Y	Y	MD	ADHD	Sb, Am	Normal	N
**18**	II-1	F	2y 9m	Brachycephaly	BC	N	N	SD	N	N	Normal	N
**19**	I-2	F	adult	normal	NA	Y	NK	+	N	Sb		N
	II-1	M	2m and 11m	Right plagiocephaly	NA	Y	Y	+	NA	Sb	N	N
	II-2^f^	M	22 week fetus	NA	NA	NA	Y	NA	NA	NA	NA	NA
**20**	I-2	F	33 years	Normal	NA	N	NK	+	N	Sb (resolved)	NK	N
	II-1	F	18 months	Severe brachycephaly, cephalic index 99%	BC	N	N	+	NA	N	Normal	N
**21**	II-1	M	7m	Turricephaly	LC	Y	Y	+	NA	Sb	Normal	N
**22**	II-2	F	30y	Plagiocephaly	NA	Y	Y	+++	ASD	ON	↑	Kyphosis, drumstick fingers

*ADHD*, attention deficit hyperactivity disorder; *AF*, anterior fontanelle; *Am*, amblyopia; *ASD*, autism spectrum disorder; *BC*, bicoronal; *BD*, brachydactyly; *CB*, cortical blindness; *Cl*, 5th finger clinodactyly; *HM*, hypermobility; *ID*, intellectual disability; *LC*, left coronal; *LL*, left lambdoid; *Me*, metopic; *MSK*, musculoskeletal; *NA*, not applicable; *NK*, not known; *ON*, optic nerve abnormality; *PS*, pansynostosis; *RE*, refractive error (myopia, hypermetropia or astigmatism); *RL*, right lambdoid; *RM*, rhizomelia; *S*, sagittal; *Sb*, strabismus; *Sc*, scoliosis; *Tc*, torticollis; *Tl*, talipes; *VA*, vertebral bone abnormality.

a Mild +, moderate ++, Severe +++, borderline +/−, SD speech delay only, MD motor delay only.

b Limited information available regarding father.

c Three deceased siblings with similar clinical features.

d Mosaic for the variant.

e Known craniosynostosis, no further clinical information available.

f Fetus terminated at 22 weeks’ gestation, preventing full determination of phenotype.

g Additional eye abnormalities detailed in Supplemental Table 3.

#### Nonsense and frameshift variants

Assessment of *ZIC1* nonsense variants suggests a different phenotypic outcome depending on position within the gene. Eleven individuals or families with nonsense variants in the final exon (exon 3) have now been identified, with craniosynostosis present in every individual (Supplemental Table 2), with the exception of the unaffected mother in family 12, who was mosaic (Table 1). We report 4 more families here, 1 of which (p.(Ser388Ter) in family 13) was described previously in an unrelated family and demonstrated to escape NMD,^7^ consistent with a possible gain-of-function mechanism. The family 11 c.1101C>A p.(Cys367Ter) variant, a de novo change identified in a child with metopic craniosynostosis, is within 55 bp of the end of exon 2 and thus also likely to escape NMD because of its proximity to the final intron. This variant was identified previously in a person with multisuture synostosis^8^ and a previous in vitro analysis of ZIC3 with a similar truncation of the C-terminal region (ZIC3 p.(Lys408Ter)) led to a significant gain-of-function in a luciferase assay.^26^ In contrast, individuals with premature termination codon variants in exon 1 have NDD without craniosynostosis (Tables 1 and 2), the phenotype consisting of intellectual disability or developmental delay together with structural brain malformations particularly affecting the cerebellum. These nonsense variants (family1: c.91C>T p.(Arg31Ter), Family 2: c.197C>A p.(Ser66Ter) and family 3: c.516C>G p.(Tyr172Ter)) are upstream of the ZF-encoding domains and are predicted to lead to NMD and loss of 1 copy of *ZIC1*. In family 3, the variant arose de novo in the apparently unaffected father, with analysis of several different tissues indicating that it was constitutional (50%) in this individual (Supplemental Figure 1). In family 1 testing of the unaffected parents was only possible in the father, who was negative for the variant. In family 2, 2 brothers inherited c.197C>A p.(Ser66Ter) from their father for whom clinical information was not available.

We report a single individual with a frameshift in exon 3 of *ZIC1*. This is the second frameshift identified in *ZIC1*^9^; these are the 2 most distally located variants identified to date, and in both cases the probands did not have craniosynostosis.

#### Missense variants

We identified 15 individuals from 12 families heterozygous for missense variants in *ZIC1*, that arose de novo in 7 cases (Table 1). Only 2 of the missense alterations were located in exon 3, comprising a recurrent variant at a CpG dinucleotide (Pro395Leu) identified in 4 unrelated families (families 17-20) in which 3 of 4 probands had craniosynostosis, and p.(Gly400Val) reported in the proband of family 21 who also had craniosynostosis. These amino acid substitutions have high CADD scores (>29) but are predicted to be likely benign (Pro395Leu) or uncertain (Gly400Val) by AlphaMissense; however, at both positions different amino acid substitutions have previously been reported in individuals with craniosynostosis.^7^^,^^12^ Penetrance of craniosynostosis with missense variants in exon 3 may be less than with exon 3 nonsense variants. Sixteen people with an exon 3 missense variant have now been described, with craniosynostosis present in 7 individuals. Those for which craniosynostosis was not reported included 2 fetuses and a 2-month-old baby, for whom the phenotype may not yet have become apparent, and also 5 adults, including parents of children with coronal synostosis (Supplemental Tables 2-4). In family 19, containing a mother and 2 children the phenotype comprised strabismus, ID/developmental delay, cerebellar malformation, and plagiocephaly, but not formally diagnosed craniosynostosis. The variant allele frequency in the mother’s blood was 40%, but further assessment of other tissues for potential mosaicism was not possible (Supplemental Table 5). In family 20 the mother did not have craniosynostosis (Table 2), whereas for the previously reported p.(Gly400Arg) variant, craniosynostosis was not a fully penetrant feature within the same family.^7^

**Figure 2:**
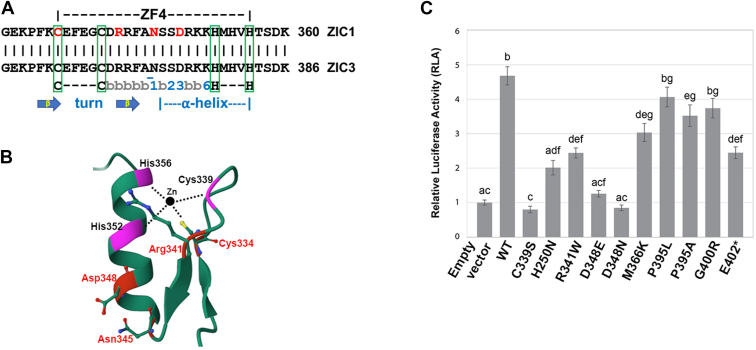
**ZIC1 structural and functional analysis.** A. Amino acid alignment of the Zn finger 4 motifs of ZIC1 and ZIC3 (structure known^6^) showing 100% identity. Residues for which missense substitutions were identified are highlighted in red. C2H2 Zn fingers consist of 2 β-strands followed by an α-helix, and the cysteine and histidine residues, which coordinate the Zn ion, are boxed in green. The residues that contact the DNA during binding are indicated as blue numbers at the −1, +2, +3, and +6 positions of the motif. B. Three-dimensional structure of the ZF4 Zn finger domain showing Zn binding and affected variants highlighted in red. C. Transactivation assay for ZIC1 substitutions. HEK293T cells were cotransfected with a ZIC reporter (B:luc2:Z3M2:β-globin)^23^ and a V5 expression construct for the indicated substitutions. Luminescence was measured 24 hours after transfection in each of 3 replicate samples and each transfection repeated 3 times. The graph shows the relative luciferase activity normalized to empty vector, such that the empty vector value becomes 1. Error bars represent the SEM. The different letters (a, b, c…) denote statistical differences, where treatment groups with the same letter are not statistically significant. As a control to confirm that luciferase activity was due to protein binding, we also used the ZIC binding site dead construct B:luc2:Z3mtt:β-globin,^23^ reducing luciferase activity by wild-type ZIC1 to that of the non-DNA-interacting mutant C339S.

The remaining 7 missense variants were located in ZIC1 ZF domains encoded by exon 1 (p.(His250Asn)) and exon 2 (p.(Cys334Gly), p.(Arg341Trp), p.(Asn345Lys,) p.(Asp348Asn), p.(Asp348Glu), p.(Met366Lys)) (Table 1) and in 5 cases had arisen de novo, with the inheritance unknown in the remaining families (families 6 and 10). All the missense variants were predicted to be likely pathogenic by AlphaMissense, had CADD scores of over 20 and were absent from gnomAD v4.1.0 except for Met366Lys (present in 2/807118 individuals; note this variant remains a VUS; Table 1). Only 3 of the 7 probands had a phenotype which included craniosynostosis (family 8, p.(Asp348Asn) and family 10, p.(Met366Lys); see below). In the 5 other probands, the phenotype consisted of facial dysmorphism, structural brain malformation, and intellectual disability or developmental delay (Table 2), and their variants were all located at conserved amino acids required for normal ZF function, either through coordination of the zinc ion or by contacting the DNA or phosphate backbone.^27^ The de novo p.(Cys334Gly) variant (family 5) affects the first cysteine of the ZF4 C2H2 motif (Figure 2A and B)^6^^,^^23^ and is predicted to lead to loss-of-function as substitution of any of the key cysteine or histidine residues completely abolishes function.^28^ The family 6 ZF4 p.(Arg341Trp) variant occurs at a phosphate backbone-contacting residue within 1 of the β-strands of the finger (Figure 2A).^6^^,^^23^ There is strong conservation of a positively charged amino acid at this position in ZF proteins,^29^^,^^30^ (Supplemental Figure 3A), and evidence that a basic residue at this position is important for nuclear transport.^31^ In silico mutagenesis suggests a new non-covalent interaction (Supplemental Figure 3B). The 3 remaining missense substitutions (p.(His250Asn), p.(Asn345Lys), and p.(Asp348Glu)) are at highly conserved residues that are known to contact the DNA strand at the key −1, +2 and +3 positions of ZFs (Figure 2A). These substitutions are predicted to alter DNA binding specificity,^25^^,^^32^ and in silico structural analysis suggests changes to the local environment and interactions (Supplemental Figures 4-7).

Two exon 1 or 2 missense variants were in families with craniosynostosis. p.(Asp348Asn) was identified in a proband with severe ID, structural brain malformations affecting the cerebellum, pons and corpus callosum, and bicoronal and sagittal synostosis (family 8, Table 2, Supplemental Table 3). The p.Asp348 is one of the residues in ZF4 that directly contacts DNA (Figure 2B), and substitution to glutamic acid was identified at the same position in family 9 (p.(Asp348Glu)), although, as described above, the proband did not have craniosynostosis. Notably, there are differences in the predicted DNA-binding sites and molecular interactions when aspartic acid or asparagine are at this position (Supplemental Figure 7), and analysis of engineered ZFs that differ only in the presence of Asp or Asn at the +3 position showed significant differences in specificity and affinity, with Asn conferring wider specificity and higher affinity^33^ (see Discussion). The p.(Met366Lys) variant, identified in the family 10 proband who had metopic craniosynostosis, is within ZF5 adjacent to the second cysteine of the C2H2 motif. In silico modeling suggests that substitution of Met366 to lysine could alter residue orientation within this key DNA interacting region (Supplemental Figure 8); however, this variant is classified as a VUS and thus has not been included in the counts of clinical features described below.

#### Inheritance

In 5 families reported here, the *ZIC1* variant was inherited from a parent, including family 12 in which the clinically unaffected mother was mosaic for c.1153G>T p.(Glu385Ter),^16^ and 2 families with the recurrent c.1184C>T p.(Pro395Leu) variant inherited from more mildly affected mothers: family 19 comprising 2 children with structural brain malformations and family 20 with a single child with craniosynostosis (Supplemental Table 5). The mother in family 19 showed a 40% variant allele fraction on next-generation sequencing, but no further investigations to assess for mosaicism were possible. In family 20, we demonstrated that the variant was de novo in the mother, and deep sequencing of a blood DNA sample revealed no evidence of mosaicism (Supplemental Figure 1). The remaining 2 inherited cases had variants in exon 1 causing a proximal premature termination codon (c.197C>A p.(Ser66Ter) in family 2 and c.516C>G p.(Tyr172Ter) in family 3. In family 3, the variant arose de novo in the apparently unaffected father, with analysis of several different tissues indicating that it was constitutional (50%) in this individual (Supplemental Figure 1). In family 2, 2 brothers inherited c.197C>A p.(Ser66Ter) from their father who was not available for clinical assessment. Three previously reported families in the literature have evidence of inheritance of their *ZIC1* variant (Supplemental Table 5): a 3 generation family in which children with craniosynostosis or DWM inherited c.1198G>C p.(Gly400Arg) from their mothers who had mild ID,^7^ a fetus with structural brain malformations who inherited c.1208C>A p.(Ser403Tyr) from their mother who was found to share the same brain malformations (no assessment of mosaicism in the mother),^10^ and 2 siblings each with p.(Pro406fs), which was not detected in the blood of either parent. No further investigations were undertaken in this family to assess whether mosaicism was confined to the germline or present in other tissues of the transmitting parent.^9^

### Functional analysis of variants in ZIC1

Selected variants were assessed in an optimized luciferase reporter assay (Figure 2C, Supplemental Figure 2), with Cys339Ser used as a readout for loss of ZIC1 transactivation ability as previously demonstrated.^23^ Function is abolished if any of the 4 residues in a ZF C2H2 motif are substituted.^28^ Substitution of ZF4 Asp348 to either glutamic acid or asparagine corresponded to Cys339Ser or empty vector output demonstrating that, in this context, there is equivalent loss of activity. Similarly, ZF residue substitutions His250Asn (ZF1) and Arg341Trp (ZF4) resulted in significant reduction in luciferase expression. The ZF5 variant Met366Lys also showed a significant reduction on transactivation (35%), although less strong. Analysis of exon 3 missense variants revealed milder effects on transactivation. The recurrently affected Pro395 and Gly400 residues are found within the evolutionary conserved Subclass A C-terminal Conserved domain, previously demonstrated to enhance the transactivation ability of ZIC3,^23^ and their substitution led to limited (Pro395Ala) or subtly reduced (though not statistically significant) transactivation (Pro395Leu, Gly400Arg). In comparison, the exon 3 truncation variant Glu402Ter that does not affect the ZF domains significantly reduced transactivation by 48%. Analysis of subcellular localization showed no difference between the wild type and variant ZIC1 (Supplemental Figure 2).

### Clinical features of individuals with heterozygous *ZIC1* variants

#### Craniofacial features

Craniosynostosis was present in 12 of 29 participants with a heterozygous pathogenic or likely pathogenic *ZIC1* variant in this study (Tables 1 and 2, Supplemental Table 3) and in 22 of 46 individuals reported to date (Supplemental Table 4). The coronal suture is most commonly affected, with 19 of 22 individuals having involvement of at least 1 coronal suture and 16 with bicoronal synostosis (Supplemental Table 6). The variants identified in individuals with craniosynostosis are predominantly located in exon 3 (19/22; 86%), and the majority of these are nonsense (12/19; 63%). Of all individuals with an exon 3 nonsense or frameshift variant 71% (12/17) had craniosynostosis; in those with a missense variant in exon 3, 44% (7/16) had craniosynostosis. Three of 8 participants with exon 2 variants had craniosynostosis, whereas no individuals with a variant in exon 1 of *ZIC1* have been found to have craniosynostosis as part of their phenotype.

At least 1 dysmorphic facial feature was identified in ∼60% of individuals with heterozygous *ZIC1* variants (17/29 this study; 28/46 total, Table 2, Supplemental Tables 3 and 4). In individuals with craniosynostosis, some of these may be attributable to their craniosynostosis, for example the flat facial profile of bicoronal craniosynostosis. Nevertheless, dysmorphic features were reported in individuals both with (76%) and without (57%) craniosynostosis, including downslanting palpebral fissures (occasionally upslanting), a high-arched palate, low set ears, abnormalities of the philtrum or upper lip, and eyebrow abnormalities (thin eyebrows and/or synophrys).

A notable feature in some individuals with heterozygous variants in *ZIC1* is deficient ossification of the skull vault. Nine individuals to date (family 17 II-1, family 20 II-1 in this study, and 7 reported previously)^7^^,^^12^ have demonstrated this feature, all with variants affecting exon 3. In the proband from family 17, left coronal and metopic synostosis is reported in conjunction with a large midline deficiency of the parietal bones consistent with giant parietal foramina, whereas individual II-1 from family 20 has widely open fontanelles and widening of the posterior sagittal suture also suggesting developing parietal foramina. The mother (I-2) from family 20 had a large fontanelle in childhood but is not known to have had craniosynostosis. Deficient skull ossification in previously reported cases included widened or delayed closure of the anterior fontanelles, bony defects of the metopic, lambdoid and sagittal sutures, and parietal bone deficiency. Together, these suggest that deficient ossification of the skull vault may be a distinctive phenotypic feature associated with heterozygous *ZIC1* variants in some individuals.

#### Structural brain malformation

**Figure 3:**
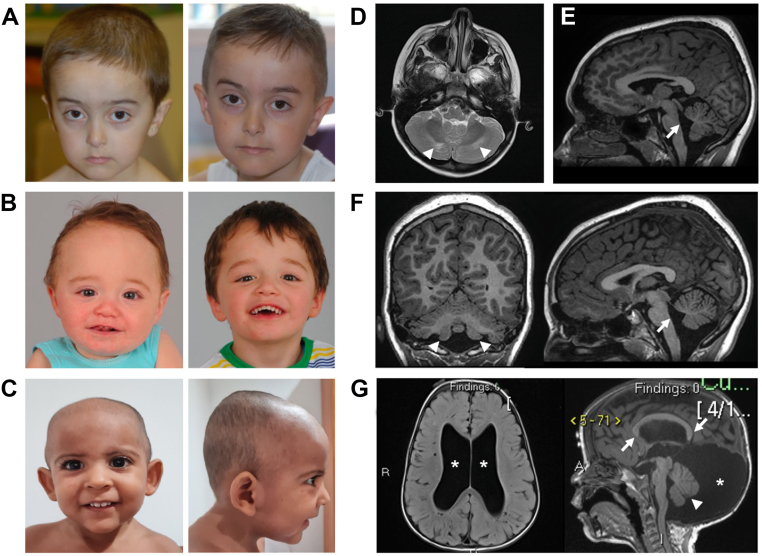
**Facial features and structural brain anomalies in individuals heterozygous for ZIC1 variants.** A. Facial appearance of proband from family 6 aged 6 years (left) and 8 years (right). B. Facial appearance of proband from family 17 aged 14 months (left) and 3 years 9 months (right). C. Facial appearance of proband from family 11 aged 19 months. D. Axial T2-weighted MRI brain section through the cerebellum of proband from family 1 aged 3 years demonstrating bilateral cerebellar hyperintensity at the gray-white matter junction (arrowheads). E and F. MRI brain images of individuals from family 2. E. Midline sagittal MRI of II-2 aged 14 years demonstrating mild cerebellar atrophy, thin brainstem and dilatation of the 4th ventricle (arrow). F. Coronal (left) and sagittal (right) MRI brain images of II-1 aged 8 years demonstrating hypoplasia and symmetrical dysplasia of the cerebellar hemispheres most evident inferiorly (arrowheads), with thin brainstem and 4th ventricle dilatation communicating with an enlarged posterior fossa (arrow). Both II-1 and II-2 also had white matter hyperintensities on MRI FLAIR imaging. G. MRI images of proband from family 7 aged 23 months. Left: Axial FLAIR MRI image demonstrates mild periventricular white matter loss, prominent perivascular spaces, and mild to moderate supratentorial ventriculomegaly with colpocephaly of the lateral ventricles (stars). Right: Sagittal T1-weighted MRI demonstrating large cyst of the posterior fossa (star), pontocerebellar hypoplasia (arrow head) and partial agenesis of the corpus callosum with hypoplasia of the rostrum and splenium (arrows).

Twenty out of 29 individuals in this study with a heterozygous *ZIC1* variant had evidence of a structural brain malformation on neuroimaging (Figure 3, Table 2, Supplemental Table 3). Brain imaging had not been performed in 7 individuals and did not show a structural brain abnormality in 2 individuals (family 18 II-1 and family 20 II-1; both harboring Pro395Leu). In those scanned, neuroimaging abnormalities were detected in 10 of 10 individuals with a variant in exons 1 or 2 of *ZIC1* (5 nonsense and 5 missense) and 10 of 12 individuals with variants in exon 3 of *ZIC1* (5 nonsense, 4 missense, and 1 frameshift). Together with previously reported cases, 33 of 46 individuals have evidence of structural brain malformations on neuroimaging. In 8 individuals, there was no reported neuroimaging, and only 5 have brain imaging reported as normal, including 3 individuals, for whom only a computed tomography of the head was available, which may be less sensitive at detecting more subtle structural brain malformations compared with a dedicated magnetic resonance imaging (MRI) scan of the brain (Supplemental Tables 3 and 4). Many features of the DWM spectrum were apparent, although only 1 individual to date^7^ has been described as having a full classic DWM. Consistent features in those examined (*n* = 37) included cerebellar dysplasia or volume loss (22), prominent or dilated cerebral ventricles commonly including but not restricted to the 4th ventricle (20), abnormalities of the corpus callosum, including hypoplasia or agenesis (16), and a thin brainstem or pontine hypoplasia (10). This common brain malformation pattern was found in individuals with and without craniosynostosis and irrespective of the location of the *ZIC1* variant. Other reported structural brain abnormalities included schizencephaly in the proband from family 4.

#### Neurological and developmental assessment

In this study, ID or global developmental delay was present in 14 of 26 individuals that were assessed; in a further 3 participants, this was recorded as borderline, and in 6 of the younger individuals, there was either motor delay or speech delay (Table 2, Supplemental Table 3). With previously reported information (Supplemental Table 4), the proportion of *ZIC1* pathogenic variant positive individuals affected by ID or global developmental delay was 66% (25/38 assessed), with 3/6 (50%) with variants in exon 1 (2 nonsense, 1 missense variant), 5/6 in exon 2 (1 nonsense, 4 missense variants), and 17/26 (65%) of individuals with exon 3 variants (5 nonsense, 9 missense, and 3 frameshift). However, in this study, many of the individuals with exon 3 variants were below the age of 5 years (including a fetus and several babies), meaning that an assessment of ID is not possible. Of the 8 individuals with exon 3 variants who were older than 5 years, 4 were thought to have ID, 2 had speech delay, 1 had mild motor delay, and 1 was a clinically unaffected parent who was mosaic (I-2 from family 12).

Variable delays in development were common even where there was not a formal ID diagnosis (Table 2, Supplemental Table 3). Here, 63% (15/24) of assessed individuals were reported to have motor delay (50% [3/6] of those with variants in exon 1, 100% with variants in exon 2 [6/6], and 50% with variants in exon 3 [6/12]), and 16 of 27 individuals had speech and language difficulties (83% [5/6] with variants in exon 1, 83% with variants in exon 2 [5/6], and 40% with variants in exon 3 [6/15]), with many of the total too young for speech delay to have become apparent. Ten individuals carrying *ZIC1* variants have a diagnosis of autism spectrum disorder (ASD) (7 in our study, and 3 previously described), comprising 2 with nonsense variants in exon 1, 4 with a nonsense or missense variant in exon 2, and 4 with an exon 3 nonsense or frameshift variant. ASD was suspected in a further individual (family 16). Four individuals reported here had attention deficit hyperactivity disorder (ADHD), 3 with a missense variant in exon 2 (families 5, 7, and 9) and 1 with a missense variant in exon 3 (family 17). ADHD has been diagnosed in 1 of the previously reported cases, with consistent features in 2 further individuals (all exon 3; Supplemental Table 4). One individual (family 9) additionally carries a pathogenic variant in *SCAF4* (1423C>T p.(Arg475Ter), HGNC:19304) inherited from his father, who had mild learning disability. The phenotype of pathogenic variants in *SCAF4* (OMIM 620511) includes ID, which is most commonly mild, and *SCAF4* variants have been reported to coexist with other genetic diagnoses.^34^ The proband in family 9, in whom the *ZIC1* variant occurred de novo, is more severely affected than his father from whom he inherited the *SCAF4* variant, having moderate learning disability for which he attends a special needs college. He additionally has cerebellar volume loss on MRI of his brain which, in contrast to *ZIC1* variants for which this is a frequent finding, is not a common feature of the *SCAF4* phenotype, including specifically for the Arg475Ter variant, which has previously been reported in an individual with mild ID and a normal MRI brain.^35^ Therefore, we consider that the *ZIC1* variant in the proband from family 9 is an important cause of his more severe ID, as part of a blended phenotype with *SCAF4*.

#### Other clinical features

Several other phenotypic features were reported in >1 individual (Table 2, Supplemental Table 3). An ocular phenotype was apparent in the participants reported here and previously, comprising strabismus in 58% of individuals (22/38) and optic nerve abnormalities in 18% (7/38). These visual features were found in association with variants throughout *ZIC1* and in individuals with and without craniosynostosis.

Abnormalities of tone were noted in 39% (9/23) of individuals assessed here, with 2 of 5 carrying exon 1 variants, 6 of 6 exon 2, and a single individual (1/12) an exon 3 variant. Five previously published individuals had abnormal tone. Where reported, low and increased tone occurred at similar frequency.

Musculoskeletal features are a common feature associated with heterozygous variants in *ZIC1*, with 47% of individuals (18/38) reported to date displaying at least 1 musculoskeletal alteration. These comprise 10 individuals with scoliosis or kyphosis, 6 individuals with joint hypermobility or joint subluxation and small numbers with other musculoskeletal abnormalities, including talipes, rocker bottom feet, and brachydactyly.

## Discussion

Previous reports have indicated that heterozygous deletions, including *ZIC1*, and the closely adjacent *ZIC4* can result in DWM,^4^ whereas isolated heterozygous variants in the final exon of *ZIC1* cause a distinct phenotype of craniosynostosis and variable ID,^7^, ^8^, ^9^, ^10^, ^11^, ^12^^,^^17^ likely due to a gain-of-function mechanism involving the C terminus.^7^ Here, we describe the largest cohort of individuals carrying *ZIC1* variants to date, confirming a syndrome including craniosynostosis, ID, and brain malformations and clarifying genotype-phenotype correlations. In particular, we provide evidence of a novel neurodevelopmental phenotype without craniosynostosis associated with proximal loss-of-function heterozygous variants in *ZIC1*.

Among the participants that we present, we identified 11 families with heterozygous truncating and deleterious missense variants of *ZIC1* exon 3, the terminal exon. Together with those already published, a total of 14 pathogenic exon 3 variants from 21 families have now been described, with the majority (62%) truncating, and craniosynostosis a major feature in 81% of families (Supplemental Table 2).^7^, ^8^, ^9^, ^10^, ^11^, ^12^^,^^16^, ^17^, ^18^ In addition 2 families with an identical exon 2 nonsense variant (c.1101C>A p.(Cys367Ter)) have now been identified (this study and^8^). This variant is predicted to truncate ZF5 and escape NMD because of its proximity with the final intron, consistent with a gain-of-function mechanism and indicating that nonsense variants at the end of exon 2 can also result in premature cranial suture fusion. In contrast, more proximal *ZIC1* variants (x9) are primarily only associated with ID and brain malformations found in previously reported cases. Thus, impaired intellectual development, cerebellar hypoplasia, corpus callosum abnormalities, and ventricular dilatation appear frequent with *ZIC1* pathogenic variants, irrespective of the location within the gene. In line with the consequences of heterozygous deletion, these features most likely arise from loss-of-function of 1 *ZIC1* allele, suggesting that the variants that also cause craniosynostosis might have both loss- and gain-of-function effects.

In addition to the *ZIC1* nonsense variants identified in exon 1 that are likely to lead to loss-of-function, several participants with missense substitutions located within ZF1 and ZF4 presented with NDD alone. Support for pathogenicity comes from their localization at key functional residues within ZF motifs (Figure 2), from in silico modeling using the solved ZIC3 structure (Supplemental Figures 3, 5-8),^6^ from the literature on known causal variants in *ZIC2* and *ZIC3*, and from our reporter assay (Figure 2C), together pointing at likely loss-of-function and/or changes to specificity.^36^ In ZIC2 and ZIC3, missense substitutions of the first cysteine in ZFs are associated with holoprosencephaly^37^ and heterotaxy,^38^^,^^39^ respectively, supporting likely pathogenicity for the c.1000T>G p.(Cys334Gly) substitution. Furthermore, functional assays of ZF4 cysteine missense variants in all ZIC paralogs demonstrate significantly reduced DNA binding in transactivation assays, including for substitution p.(Cys339Ser) in ZIC1 (Figure 2C).^23^ The ZF4 Arg341 residue contacts the phosphate backbone making a key contribution to DNA binding strength and in our functional assay this change resulted in significantly reduced transactivation. The remaining missense changes which all arose de novo were located within ZF motifs at key DNA-contacting positions -1 (p.(Asn345Lys)), +2 (p.(His250Asn)) and +3 (p.(Asp348Glu/Asn)). In silico modeling of these substitutions predicted changes to the local structural environment, and a substitution at the same position as Asn345 in ZIC3 was previously characterized as likely pathogenic, with a functional assay suggesting partial loss-of-function.^40^ Functional assessment of substitutions of His250 and Asp348 suggests that they lead to loss-of-function, with a reduction in transactivation ability similar to the loss of DNA-binding mutant p.(Cys339Ser) (Figure 2C). These missense changes could also affect specificity, not assessable in our assay, but in relation to p.(Asp348Glu) the reverse change from glutamate to aspartate at position +3 of the KLF1 (HGNC:6345) ZF2 in the neonatal anemia (*Nan*) mouse altered DNA binding specificity resulting in a phenotype that was more severe than loss-of-function because of the reorganization of interactions between DNA-binding domain residues.^41^ Considering this, it is interesting that substitution of the same residue to Asn (family 8) was in addition associated with bicoronal and sagittal craniosynostosis (Table 2, Supplemental Table 6), suggesting mechanistic differences between these 2 substitutions.

Although the majority of *ZIC1* variants appear to arise de novo, familial cases have been identified,^7^^,^^9^, ^10^, ^11^ including 5 families in this study. In family 12, the unaffected mother was mosaic for the variant, whereas in families 19 and 20, the mothers had a history of strabismus and mild learning problems and were more mildly affected than their children; assessment of mosaicism in family 20 was negative. Of note, the 2 remaining inherited cases both involved nonsense variants in exon 1 (p.(Ser66Ter) and p.(Tyr172Ter)). In one, the father, deduced to be constitutionally heterozygous based on analysis of 3 different tissues, was clinically unaffected, whereas in the other, it was noted that the father had a history of behavioral problems, but no further information was available, and the possibility of somatic mosaicism was not investigated. We note that there are 10 potential loss-of-function *ZIC1* variants in gnomAD v4.1.0 (all exon 1), but only 2 of these are likely heterozygous loss-of-function variants (Supplemental Table 7). Nevertheless, these cases support that heterozygous *ZIC1* variants do not always lead to clinically apparent features and illustrate phenotypic variability and the need for careful clinical and molecular assessment of an apparently unaffected parent. Mosaicism has been detected in 3 individuals (out of 33 families) reported to date (Supplemental Table 5) and not investigated in many others. This necessitates careful counseling regarding recurrence risk, and consideration of assessment of parental somatic mosaicism in families in which the variant is not detected in parental blood because of the potential for unrecognized gonasomal mosaicism. Interestingly, there is an uncharacterized alternative *ZIC1* transcript (Ensembl ENST00000488404), which is lacking exon 1 and is more highly expressed in the cerebellum than the canonical transcript (https://gtexportal.org/home/gene/ZIC1#gene-transcript-browser-block). Variable penetrance of exon 1 stop variants could perhaps occur through the relative expression of the wild-type allele in conjunction with the alternative splicing balance.

Heterozygous deletions of *ZIC1* together with *ZIC4* are associated with the posterior fossa abnormality DWM, characterized by hypoplasia of the cerebellar vermis and dilatation of the 4th ventricle, although penetrance is not complete.^4^ Only 1 of the previously reported cases with *ZIC1* SNVs has shown DWM,^7^ and 2 ∼1-Mb deletions involving *ZIC1/ZIC4* are reported in DECIPHER,^42^ both with phenotypes including ID and developmental delay, but not DWM. However, a common feature in our participants is structural brain malformations including the cerebellum and ventricles, without the full DWM phenotype, with cerebellar hypoplasia, ventricular dilatation, and hypoplasia or agenesis of the corpus callosum detected. These findings are not correlated with variant location or type, suggesting that they occur through loss of normal *ZIC1* function. Supportive of this, mice heterozygous for *Zic1* loss show abnormal cerebellar development and homozygous mice have cerebellar hypoplasia.^1^

Many individuals, both with and without craniosynostosis, had developmental delays or ID, with speech and language delay, motor delay, and ASD or ADHD being common. This may not yet be apparent in several of the cases because of their young age. Nevertheless, severity of learning problems appears variable, from relatively mild to severe, and there was no clear correlation between location of the variant and severity of the neurodevelopmental phenotype. The cooccurrence between coronal synostosis and ID is rare, and *ZIC1* should be particularly considered when these 2 features coincide.

Among other reported manifestations, sleep disturbance, abnormalities of tone, and musculoskeletal phenotypes were recorded. Some individuals had hypotonia, whereas others are reported to have increased tone, clonus, or spasticity. The most common musculoskeletal manifestations were scoliosis and other abnormalities of the vertebral column. Progressive scoliosis has been previously reported in 2 individuals carrying a *ZIC1* pathogenic variant.^7^ Notably, mice deficient in *Zic1* have vertebral and rib defects, and Zic1-lineage stem cells have recently been shown to play a key role in the formation and maintenance of vertebral bone.^43^

The most commonly involved suture in individuals with *ZIC1* variants is the coronal suture, with or without metopic, lambdoid, or sagittal suture involvement (Supplemental Table 6). This may reflect the specific developmental origin of the coronal suture and the high genetic load in coronal craniosynostosis.^44^ Moreover, *Zic1* is expressed at the coronal suture during formation and growth,^7^^,^^14^^,^^15^ but the role of *ZIC1* in suture development is not understood. In contrast, noncoronal suture fusion has only been found twice, in individuals with isolated metopic craniosynostosis and more proximal variants (p.Met366Lys and p.Cys367Ter) affecting adjacent residues in ZF5, who also had significant structural brain malformations. The isolated metopic synostosis in these individuals could be secondary to the underlying brain malformations, and p.Met366Lys is currently considered a VUS.

In summary, we have presented the largest cohort of individuals to date who carry monoallelic variants in *ZIC1*. Large-scale analyses for de novo pathogenic variants in NDDs have not identified a significant enrichment of variants in *ZIC1*.^45^, ^46^, ^47^ Here, we demonstrate a core phenotype of structural brain malformations, dysmorphic features, and developmental delay/ID with presence or absence of craniosynostosis largely being determined by the location of the *ZIC1* variant. This dichotomous pattern is reminiscent of the recent finding that somatic *ZIC1* pathogenic variants in G4 (loss of function) versus Sonic Hedgehog (gain of function, C-terminal) medulloblastoma are context dependent.^48^ Although the usual outcome of C-terminal *ZIC1* variants is craniosynostosis through a proposed gain-of-function mechanism, describe a neurodevelopmental phenotype without craniosynostosis due to proximal loss-of-function variants.

## Data Availability

All data are available in the manuscript and supplemental material.

## Conflict of Interest

The authors declare no conflicts of interest.
