# Supplementary material for: Expanding the phenotypic spectrum associated with ZIC1 variants: A neurodevelopmental disorder with and without craniosynostosis
Source: Genet Med. 2026 Jun;28(6):102585. doi: 10.1016/j.gim.2026.102585 (PMC13328068; doi:10.1016/j.gim.2026.102585)
Supplement: Supplemental Tables 1-7 and Figures 1-8 [file mmc1.pdf]

Supplemental Information

Supplemental Table 1 ACMG classification criteria applied and evidence used
Supplemental Table 2 *ZIC1* variants described in this study and previously
Supplemental Table 3 Clinical details of families in this study (Excel file)
Supplemental Table 4 Clinical details of previously reported cases (Excel file)
Supplemental Table 5 Summary of families where the *ZIC1* variant was inherited or present in a mosaic state
Supplemental Table 6 Patterns of suture fusion in individuals heterozygous for functionally significant *ZIC1* variants
Supplemental Table 7 Loss-of-function variants in *ZIC1* from gnomAD

Supplemental Figure 1 Families 3 & 20: segregation analysis
Supplemental Figure 2 ZIC1 transactivation assays. Individual replicates with representative western blot
Supplemental Figure 3 Sequence conservation of ZF4 and in silico structural analysis of p.Arg341Trp
Supplemental Figure 4 Predicted binding specificity changes for ZIC1 zinc finger substitutions
Supplemental Figure 5 p.His250Asn in silico structural analysis and conservation
Supplemental Figure 6 In silico structural analysis of p.Asn345Lys
Supplemental Figure 7 p.Asp348Glu in silico structural analysis and conservation
Supplemental Figure 8 p.Met366Lys in silico structural analysis and conservation

Supplemental Table 1 ACMG classification criteria applied and evidence used

				Population data		Computational and predictive data				Functional data		De novo data	Other data		
Exon	HGVS (NC_000003.12) GRCh38	cDNA (NM_003412.4)	Protein (NP_003403.2)	PM2: gnomAD prevalence (v4.1.0)	PS4: Previous cases[a]	PVS1: Null variant with LOF mechanism of disease[b]	PM4: Final exon truncating variant with GOF mechanism of disease[c]	PM5: Missense at residue where different missense pathogenic	PP3: Computational evidence	PM1: Hot spot/functional domain<br><br>PP2: missense constraint	PS3: Functional assay[f]	PS2 (confirmed parentage)/PM6	PP4: Highly specific phenotype[g]	Criteria	Classification
1	g.147410203C>T	c.91C>T	p.(Arg31Ter)	0	-	+			CADD 35	-	Not tested	Unknown		PM2, PVS1 (strong)	LP
1	g.147410309C>A	c.197C>A	p.(Ser66Ter)	0	-	+			CADD 36	-	Not tested	N		PM2, PVS1 (strong))	LP
1	g.147410628C>G	c.516C>G	p.(Tyr172Ter)	0	-	+			CADD 36	-	Not tested	De novo in father		PM2, PVS1 (strong), PM6	LP
1	g.147410860C>A	c.748C>A	p.(His250Asn)	0	-				CADD 26.8, AM 0.915	DNA contacting ZF residue, altered in silico analysis	Reduced transactivation	De novo		PM2, PP3, PS3, PM6,	LP
2	g.147412535T>G	c.1000T>G	p.(Cys334Gly)	0	-				CADD 32, AM 0.998	Canonical cysteine of ZF C2H2 motif[e]	Not tested	De novo		PM2, PP3, PM1 (strong), PM6	LP
2	g.147412556C>T	c.1012C>T	p.(Arg341Trp)	0	-				CADD 31, AM 0.993	Phosphate backbone contacting, in silico modelling	Reduced transactivation	Unknown		PM2, PP3, PS3	LP

										suggests new interaction					
2	g.147412570C >G	c.1035C>G	p.(Asn345L ys)	0	-				CADD 24, AM 0.999	DNA contacting, altered in silico modelling	Not tested	De novo		PM2, PP3, PM1, PM6	LP
2	g.147412577G >A	c.1042G>A	p.(Asp348A sn)	0	-				CADD 32, AM 0.995		Reduced transactivati on	De novo (trio exome)		PM2, PP3, PS3, PS2	P
2	g.147412579C >G	c.1044C>G	p.(Asp348G lu)	0	-				CADD 23, AM 0.992		Reduced transactivati on	De novo		PM2, PP3, PS3, PM6	LP
2	g.147412632T> A	c.1097T>A	p.(Met366L ys)	0.000001 239	Not in literatu re, 3 in ClinVar-VUS-no report of clinical feature s				CADD 25.4, AM 0.6051	Altered in silico modelling	Reduced transactivati on	Unknown		PP3, PS3	VUS
2	g.147412636C >A	c.1101C>A	p.(Cys367T er)	0	Miller et al., 2017 & 2 reports in ClinVar				CADD 37			De novo		PM2, PS4 (moderat e), PP3, PM6	LP
3	g.147413360G >T	c.1153G>T	p.(Glu385T er)	0	-		+		CADD 25.2		Not tested	De novo (trio)		PM2, PM4, PP3, PS2	LP
3	g.147413370C >A	c.1163C>A	p.(Ser388T er)	0	Twigg et al., 2015 (1)		+		CADD 31		Not tested	De novo in Twigg et al., 2015		PM2, PS4 (supporti ng), PM4, PP3, PM6	LP
3	g.147413381C >T	c.1174 C>T	p.(Gln392T er)	0	-		+		CADD 45		Not tested	De novo		PM2, PM4, PP3, PM6	LP

3	g.147413388C>A	c.1181C>A	p.(Ser394Ter)	0	2 unrelated cases reported here		+		CADD 39		Not tested	Unknown	+	PM2, PS4 (supporting), PM4, PP3, PP4	LP
3	g.147413391C>T	c.1184C>T	p.(Pro395Leu)	0	4 unrelated Pro395Leu families reported here			p.(Pro395Ala) (Sasaki et al., 2020)[d]	CADD 29.5, AM 0.197	Missense constrained region	Not significant	In 2/4 families		PM2, PS4 (moderate), PM5 (supporting), PP2, PM6	LP
3	g.147413406G>T	c.1199G>T	p.(Gly400Val)	0				p.(Gly400Arg) reported by Twigg et al., 2015 in 5 affected individuals of a 3 generation family	CADD 29.2, AM 0.466	Missense constrained region	Not tested	Unknown	+	PM2, PM5, PP2,PP4	LP
3	g.147413414_147413415insA	c.1207_1208insA	p.(Ser403TyrfsTer41)	0	-	+			-		Not tested	De novo		PM2, PVS1 (moderate), PM6	LP

ACMG: American College of Medical Genetics; AM: AlphaMissense score; LOF: loss of function; GOF: gain of function; ZF: zinc finger; P: pathogenic; LP: likely pathogenic.

Variants were classified using the Association for Clinical Genomic Science (ACGS) Best Practice Guidelines for Variant Classification in Rare Disease (https://www.acgs.uk.com/media/12533/_media_12533_uk-practice-guidelines-for-variant-classification-v12-2024.pdf), which provide additional guidance for interpretation of the 2015 ACMG criteria.

[a]PS4 was applied at a moderate level if the variant had been previously identified in two or more unrelated individuals, or at a supporting level if identified in one individual previously with a rare and specific phenotype.

[b]PVS1 was applied to exon 1 nonsense variants according to specific supplemental guidance for interpretation of the loss of function PVS1 criterion, where the strength with which this is applied depends upon the evidence of a loss of function phenotype (from 3q25 deletions and null mouse models), whether the transcript is predicted to undergo NMD and the proportion and domains of the protein affected. PVS1 was not used together with PM1, PM4, PP2, PP3.

[c]Nonsense and truncating variants in the final exon were interpreted according to Figure 1 of the ACGS guidance, with PM4 applied for nonsense variants based upon previous evidence of gain of function, and PVS1 applied at reduced weighting for the final exon frameshift variant p.(Ser403Tyrfs*41).

[d]PM5 applied at supporting level as appropriate for a single previous report of a likely pathogenic variant.
[e]PM1 weight increased to strong for this variant affecting a canonical cysteine of the C2H2 zinc finger motif.
[f]PS3 was applied based upon the results of the luciferase assays reported here.
[g]Applied for the specific phenotypic combination of coronal synostosis with intellectual disability, facial dysmorphism and structural brain abnormality, where phenotypic specificity has not already been captured in PS2/PM6.

Supplemental Table 2 *ZIC1* variants described in this study and previously

Exon	HGVS (NC_000003.12)	cDNA (NM_003412.4)	Protein (NP_003403.2)	De novo?	AM[a] score[a]	CRS	Source[b]
1	g.147410203C>T	c.91C>T	p.(Arg31Ter)	unknown	-	-	This study(1)
1	g.147410309C>A	c.197C>A	p.(Ser66Ter)	N	-	-	This study(2)
1	g.147410628C>G	c.516C>G	p.(Tyr172Ter)	+ (in father)	-	-	This study(3)
1	g.147410860C>A	c.748C>A	p.(His250Asn)	+	0.915	-	This study(4)
2	g.147412535T>G	c.1000T>G	p.(Cys334Gly)	+	0.9979	-	This study(5)
2	g.147412556C>T	c.1012C>T	p.(Arg341Trp)	unknown	0.9934	-	This study(6)
2	g.147412570C>G	c.1035C>G	p.(Asn345Lys)	+	0.999	-	This study(7)
2	g.147412577G>A	c.1042G>A	p.(Asp348Asn)	+	0.9947	+	Timberlake et al., 2023 & this study(8)
2	g.147412579C>G	c.1044C>G	p.(Asp348Glu)	+	0.9917	-	This study(9)
2	g.147412632T>A	c.1097T>A	p.(Met366Lys)	unknown	0.6051	+	This study(10)
2	g.147412636C>A	c.1101C>A	p.(Cys367Ter)	+	-	+	This study(11)
2	g.147412636C>A	c.1101C>A	p.(Cys367Ter)	suspected	-	+	Miller et al., 2017
3	g.147413360G>T	c.1153G>T	p.(Glu385Ter)	+ Mother mosaic	-	+	Tonne et al., 2021 & this study(12)
3	g.147413370C>A	c.1163C>A	p.(Ser388Ter)	+	-	+	Twigg et al., 2015
3	g.147413370C>A	c.1163C>A	p.(Ser388Ter)	AD	-	+	This study(13)
3	g.147413372C>T	c.1165C>T	p.(Gln389Ter)	+	-	+	Twigg et al., 2015
3	g.147413372C>T	c.1165C>T	p.(Gln389Ter)	+	-	+	Chen et al., 2022
3	g.147413379C>A	c.1172C>A	p.(Ser391Ter)	unknown	-	+	Bukowska-Olech et al., 2022
3	g.147413381C>T	c.1174 C>T	p.(Gln392Ter)	+	-	+	This study(14)
3	g.147413388C>A	c.1181C>A	p.(Ser394Ter)	unknown	-	+	This study(15)
3	g.147413388C>A	c.1181C>A	p.(Ser394Ter)	unknown	-	+	This study(16)
3	g.147413390C>G	c.1183C>G	p.(Pro395Ala)	+	0.094	+	Sasaki et al., 2020
3	g.147413391C>T	c.1184C>T	p.(Pro395Leu)	unknown	0.1969	+	This study(17)
3	g.147413391C>T	c.1184C>T	p.(Pro395Leu)	+	0.1969	+	This study(18)
3	g.147413391C>T	c.1184C>T	p.(Pro395Leu)	AD	0.1969	-	This study(19)
3	g.147413391C>T	c.1184C>T	p.(Pro395Leu)	+	0.1969	+	This study(20)
3	g.147413405G>C	c.1198G>C	p.(Gly400Arg)	AD	0.8003	+	Twigg et al., 2015
3	g.147413406G>T	c.1199G>T	p.(Gly400Val)	unknown	0.4664	+	This study(21)
3	g.147413411G>T	c.1204G>T	p.(Glu402Ter)	+	-	+	Twigg et al., 2015
3	g.147413411G>T	c.1204G>T	p.(Glu402Ter)	+	-	+	Twigg et al., 2015
3	g.147413415C>A	c.1208C>A	p.(Ser403Tyr)	AD	-	-	Pangalos et al., 2016
3	g.147413414_147413415insA	c.1207_1208insA	p.(Ser403TyrfsTer41)	+	-	-	This study(22)

3	g.147413421_147413422insCACCATCGTG	c.1214_1215insCACCATCGTG	p.(Pro406fsThrfsTer41)	Gonadal mosaicism	-	-	Vandervore et al., 2018

[a]AM score – AlphaMissense scores (ref: Cheng et al. 2023. *Science* DOI: 10.1126/science.adg7492): red, pathogenic ; green, benign; blue, ambiguous.
[b]Family number in brackets for this study. CRS, craniosynostosis; N, no; AD, autosomal dominant.

Supplemental Table 5: Summary of families where the *ZIC1* variant was inherited or present in a mosaic state

Reference	Family	HGVS (NC_000003.12)	cDNA (NM_003412.4)	Amino acid change (NP_003403.2)	Individual	Major clinical features	Assessment of mosaicism
This study	2	g.147410309C>A	c.197C>A	p.(Ser66*)	I-1	NK	NK
					II-1	SBM, speech and motor delay	
					II-2	SBM, speech delay	
This study	3	g.147410628C>G	c.516C>G	p.(Tyr172*)	II-1	Clinically unaffected	Assessment of urine, saliva and blood indicates constitutional (~50% allele frequency)
					II-1	Dysmorphism, SBM, ID	
This study and Tonne et al., 2021	12	g.147413360G>T	c.1153G>T	p.(Glu385*)	I-2	Clinically unaffected	Mosaic, 20% variant allele in blood
					II-2	CRS, dysmorphism, SBM, speech delay, strabismus, optic nerve hypoplasia, scoliosis	
This study	19	g.147413391C>T	c.1184C>T	p.(Pro395Leu)	I-2	Dysmorphism, strabismus, mild learning disability	40% variant allele in blood (NGS, 112 reads). Considered in heterozygous range, no further assessment.
					II-1	Dysmorphism, SBM, strabismus, developmental delay	
					II-2[a]	SBM	
This study	20	g.147413391C>T	c.1184C>T	p.(Pro395Leu)	I-2	Mild ID, strabismus, large fontanelle in childhood	
					II-1	CRS, deficient skull ossification, borderline developmental delay	
Twigg, 2015	3	g.147413411G>T	c.1204G>T	p.(Glu402*)	Participant 3	CRS, bony defect sagittal suture	Mosaic, variant present in 34% of reads in blood, but not evident in DNA from scalp fibroblasts
Twigg, 2015	5	g.147413405G>C	c.1198G>C	p.(Gly400Arg)	5:II.2	SBM, mild ID, delayed closure AF	Six clinically affected individuals across 3 generations. Variant present in two mildly affected siblings (5:II.2 and 5:II.4) who

							each transmitted to affected children. No genetic analysis in the clinically affected parent of 5:II.2 and 5:II.4.
					5:II.4	SBM, strabismus, mild learning difficulties	
					5:III.1	SBM, mild ID, delayed closure AF	
					5:III.3	CRS, bony defect lambdoid sutures, strabismus, mild ID	
					5:III.6	CRS, parietal foramina, SBM, strabismus, mild learning difficulties	
Pangalos, 2016		g.147413415C>A	c.1208C>A	p.S403Y	Mother	Dysmorphism, SBM	Present in maternal blood, no further assessment of mosaicism
					Fetus	SBM[a]	
					2 further fetuses share the same SBM, no genetic evaluation available		
Vandervore, 2018		g.147413421_147413422insCACCATCGTG	c.1214_1215insCACCATCGTG	p.(Pro406fsThrfsTer41)	Sibling 1	Dysmorphism, SBM, ID, strabismus, cortical visual impairment, scoliosis, abnormal tone	Absent in blood of both parents, germline mosaicism
					Sibling 2	Delayed cranial suture fusion, dysmorphism, SBM, ID, strabismus, optic nerve hypoplasia, scoliosis, abnormal tone	

SBM structural brain malformation; ID intellectual disability; CRS craniosynostosis; AF anterior fontanelle.

[a]fetus, preventing further clinical assessment.

Supplemental Table 6. Patterns of suture fusion in individuals heterozygous for functionally significant *ZIC1* variants

	Protein (NP_003403.2)	p.(Asp348Asn)[a]	p.(Met366Lys)	p.(Cys367*)	p.(Cys367*)[b]	p.(Glu385*)[c]	p.(Ser388*)	p.(Ser388*)	p.(Ser388*)[d]	p.(Gln389*)[d]	p.(Gln389*)[e]	p.(Ser391*)[f]	p.(Gln392*)	p.(Ser394*)	p.(Ser394*)	p.(Pro395Ala)[g]	p.(Pro395Leu)	p.(Pro395Leu)	p.(Pro395Leu)	p.(Gly400Arg)[d]	p.(Gly400Arg)[d]	p.(Gly400Val)	p.(Glu402*)[d]	p.(Glu402*)[d]	Total
	**Family #**	**8**[a]	**10**	**11**	16[b]	**12**[c]	**13**	**13**	1[d]	4[d]	W033[e]	129[f]	**14**	**15**	**16**[g]	-[h]	**17**	**18**	**20**	5[d]	5[d]	**21**	2[d]	3[d]	
Suture(s) fused	Metopic		X	X[i]						*		X			X		X		*						**5**
	Left Coronal	X				X	X	X	X	X	X		X		X	X	X	X	X	X	X	X	X	X	**18**
	Right Coronal	X				X	X	X	X	X	X		X		X	X		X	X	X	X		X	X	**16**
	Unicoronal											X					X					X			**3**
	Sagittal	X			X					*		X			X				*					*	**4**
	Left Lambdoid					X							X		X					*					**3**
	Right Lambdoid									X					X					*					**2**
	Unilambdoid					X				X		X	X												**4**
	Multi-suture	X			X	X	X	X	X	X	X	X	X		X	X	X	X	X	X	X		X	X	**19**
	Unknown													X											**1**
**HGVS (NC_000003.12); cDNA (NM_003412.4)**		g.147412577G>A;c.1042G>A	g.147412632T>A; c.1097T>A	g.147412636C>A; c.1101C>A	g.147412636C>A; c.1101C>A	g.147413360G>T; c.1153G>T	g.147413370C>A; c.1163C>A	g.147413370C>A; c.1163C>A	g.147413370C>A; c.1163C>A	g.147413372C>T; c.1165C>T	g.147413372C>T; c.1165C>T	g.147413379C>A; c.1172C>A	g.147413381C>T; c.1174 C>T	g.147413388C>A; c.1181C>A	g.147413388C>A; c.1181C>A	g.147413390C>G; c.1183C>G	g.147413391C>T; c.1184C>T	g.147413391C>T; c.1184C>T	g.147413391C>T; c.1184C>T	g.147413405G>C; c.1198G>C	g.147413405G>C; c.1198G>C	g.147413406G>T; c.1199G>T	g.147413411G>T; c.1204G>T	g.147413411G>T; c.1204G>T	

[a]Timberlake et al., 2023 and this study; [b]Miller et al.,2017; [c]Tonne et al., 2021 and this study ; [d]Twigg et al., 2015; [e]Chen et al., 2021; [f]Bukowska-Olech et al., 2022; [g]Pansynostosis; [h]Sasaki et al., 2020 ; [i]trigonocephaly; *bony defect of indicated suture

Supplemental Table 7 Loss-of-function variants in *ZIC1* from gnomAD (v4.1.0)

Variant ID_Chr3	HGVS (NC_000003.12)	cDNA (NM_003412.4)	Exon	ZIC1 Prediction	Allele count	Context	Data source	Variant reads
147393925-G-GC	g.147393925G>GC	N/A	?	p.(Gln12ProfsTer15)	2/456742	Variant is within an intron of ZIC4	Exome	19/46; 19/36
147410134-C-T	g.147410134C>T	c.22C>T	1	p.(Gln8Ter)	1/1587292	C homo-polymer	Exome	35/75
147410138-AC-A	g.147410139del	c.29del	1	p.(Pro10GlnfsTer5)	1/1590382	C homo-polymer	Exome	3/12
147410429-GT-G	g.147410430del	c.321del	1	p.(Phe107LeufsTer57)	1/1603748	T homo-polymer	Exome	6/18
147410487-CG-C	g.147410488del	c.380del	1	p.(Gly127AlafsTer37)	2/1606086	G homo-polymer	Exome	3/12
147410496-CG-C	g.147410497del	c.389del	1	p.(Gly130AlafsTer34)	2/1605954	G homo-polymer	Exome	3/12
147410646-C-A	g.147410646C>A	c.534C>A	1	p.(Tyr178Ter)	1/1613678	Double nucleotide variant	Exome	codon TAC>TCA: p.(Tyr178Ser)– missense: 1/1613660 in gnomAD.
147410812-C-T	g.147410812C>T	c.700C>T	1	p.(Gln234Ter)	1/1614132		Genome	12/36 (33%)
147410863-G-T	g.147410863G>T	c.751G>T	1	p.(Glu251Ter)	1/1614266		Genome	46/94
147411095-G-T	g.147411095G>T	c.982+1G>T	Intron 1 donor	c.982+1G>T	1/1610794		Genome	15/43 (35%)

Supplemental Figure 1A,B. Family 3: segregation of the c.516C>G variant (p.Tyr172Ter)

[HGVS NC_000003.12:g.147410628C>G; NM003412.4:c516C>G]

A **Initial segregation analysis**

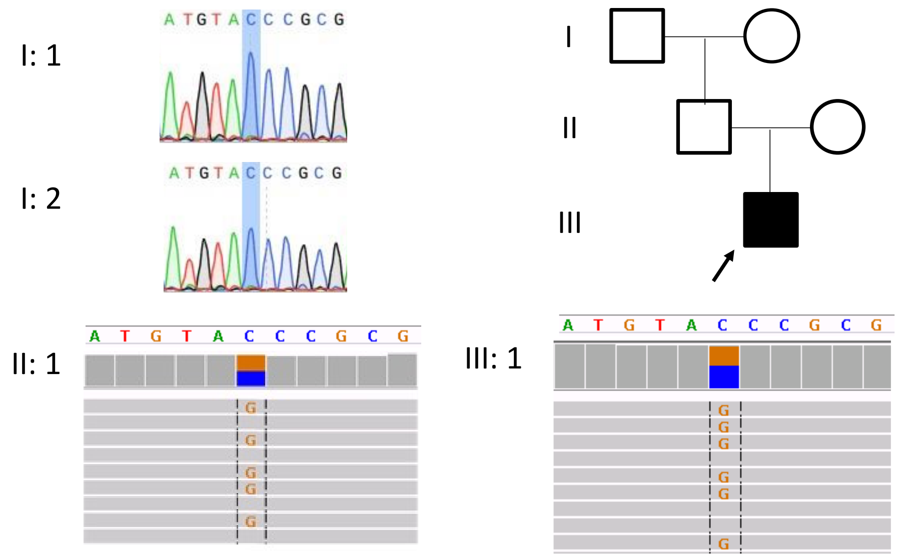

B **Deep next-generation sequencing**

	Saliva	Urine	Blood
II: 1	7194/14358 (50.1%)<br>3970/7769 (51.1%)<br>5365/11705 (45.8%)	(10030/19364) 51.8%<br>(6061/12040) 50.3%<br>(7799/16564) 47.1%	5544/11635 (47.6%)<br>5614/10894 (51.5%)<br>3652/7645 (47.8%)
	**49.0%** (+/- 2.8%)	**49.7%** (+/- 2.4%)	**49.0%** (+/- 2.2%)

A: Pedigree and segregation analysis. The paternal grandparents (I:1 & I:2) chromatograms are homozygous for the reference allele. The proband (III-1) and father's (II-1) heterozygous genotypes were determined through the 100,000 genomes project, and a representation of the sequencing read allele split is shown.

B: Summary of the deep sequencing data using different samples from the father (II-1) . Each sample has the number of variant allele reads and frequency for each technical replicate, as well as the overall weighted average of these, and an error percentage of one standard deviation. Deep sequencing of the proband (III-1; blood sample) demonstrated the same AF as his father (data not shown)

Methods:

The Family 3 proband with a paternally inherited p.Tyr172Ter variant was identified in 100,000 genomes data. The proband and father are both heterozygous, with a roughly 50:50 split of individual reads as shown in part A. Following clinical collaboration request, consent was obtained for this family to be enrolled in the 'Genetics of Craniofacial Malformations' study. A blood-derived DNA sample for the father was provided by Chapel Allerton Hospital Leeds, and blood-derived DNA for both paternal grandparents was provided by the Churchill Hospital Oxford.

Primers were designed for chr3:147410414-147411111: ZIC1 Tyr172Ter F =TGTTCCGCAACCGGGGTTTT ZIC1 Tyr172Ter R =TACAGCTGTTTCCGTACCTGTG. These were used for a Roche FastStart Taq DNA Polymerase PCR with the paternal grandparent samples, and Sanger sequenced with the reverse primer. Both parental grandparents were homozygous for the reference allele.

To determine whether the father's *de novo* variant was post-zygotic or constitutive, samples of urine and saliva for the proband's father were provided by Chapel Allerton Hospital Leeds. DNA was extracted from the saliva and urine using a Zymo Bioscience Quick-DNA Midiprep Plus Kit (D4075). Q5 High-Fidelity DNA polymerase was used for a PCR with primers targeting chr3:147410559-147410779, with a CS1 & CS2 tag appended to the forward and reverse primer respectively (CS1-ZIC1 F = acactgacgacatGGTTCTACACCACGCGTCGCCTAACGTGG & CS2-ZIC1 R= tacggtagcagagacttggtctGCTTGATGGGTTGGCGCATGTAGC), with 3 technical replicates for each sample type. The target amplicon was electrophoretically separated using a 2% agarose gel and extracted with an NEB Monarch DNA Gel Extraction Kit (T1020G). The target amplicon was subsequently barcoded with Fluidgm Access Array barcodes (100-3771) using iProof enzyme (172-5301). These libraries were pooled and purified using AMPure XP beads (A63880) at 0.8X beads/PCR products. An Illumina Miseq platform was used to generate deep next-generation sequencing data as described in Bernkopf *et al.* 2017, and this was analysed using the amplimap program (Koelling *et al.* 2019). The raw sequence was aligned using Burrows-Wheeler aligner, and this was aligned to hg38 (Li et al. 2009). The analysed data were consistent with this being a constitutive *de novo* variant in the father (II-1).

References:

Bernkopf M, Hunt D, Koelling N, Morgan T, Collins AL, Fairhurst J, et al. Quantification of transmission risk in a male patient with a FLNB mosaic mutation causing Larsen syndrome: Implications for genetic counseling in postzygotic mosaicism cases. Human Mutation. 2017;38(10):1360-4.
Koelling N, Bernkopf M, Calpena E, Maher GJ, Miller KA, Ralph HK, et al. amplimap: a versatile tool to process and analyze targeted NGS data. Bioinformatics. 2019;35(24):5349-50.
Li H, Durbin R. Fast and accurate short read alignment with Burrows-Wheeler transform. Bioinformatics. 2009;25(14):1754-60.

Supplemental Figure 1C. Family 20: segregation of the c.1184C>T variant (p.Pro395Leu)

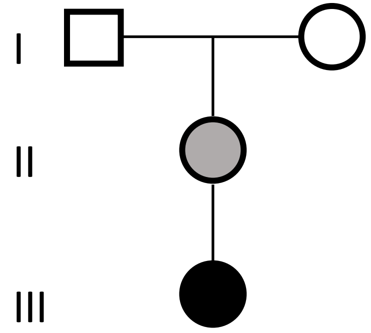

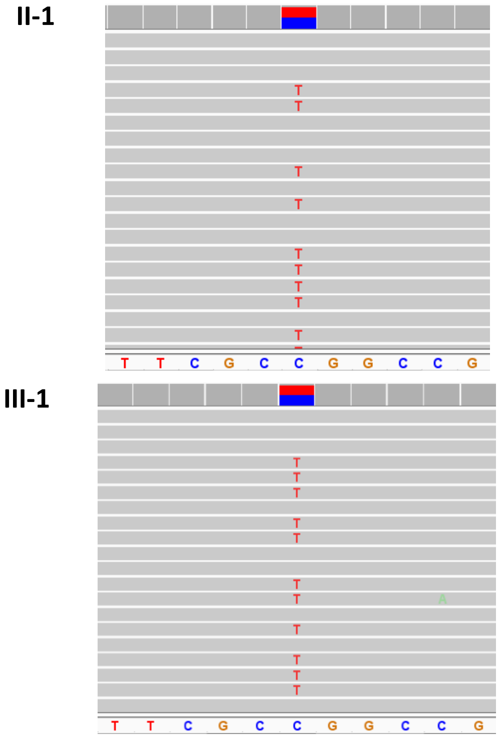

I-1	0.01%	blood
I-2	0.01%	saliva
II-1	49.02%	blood
III-1	49.60%	blood
control	0.03%	blood

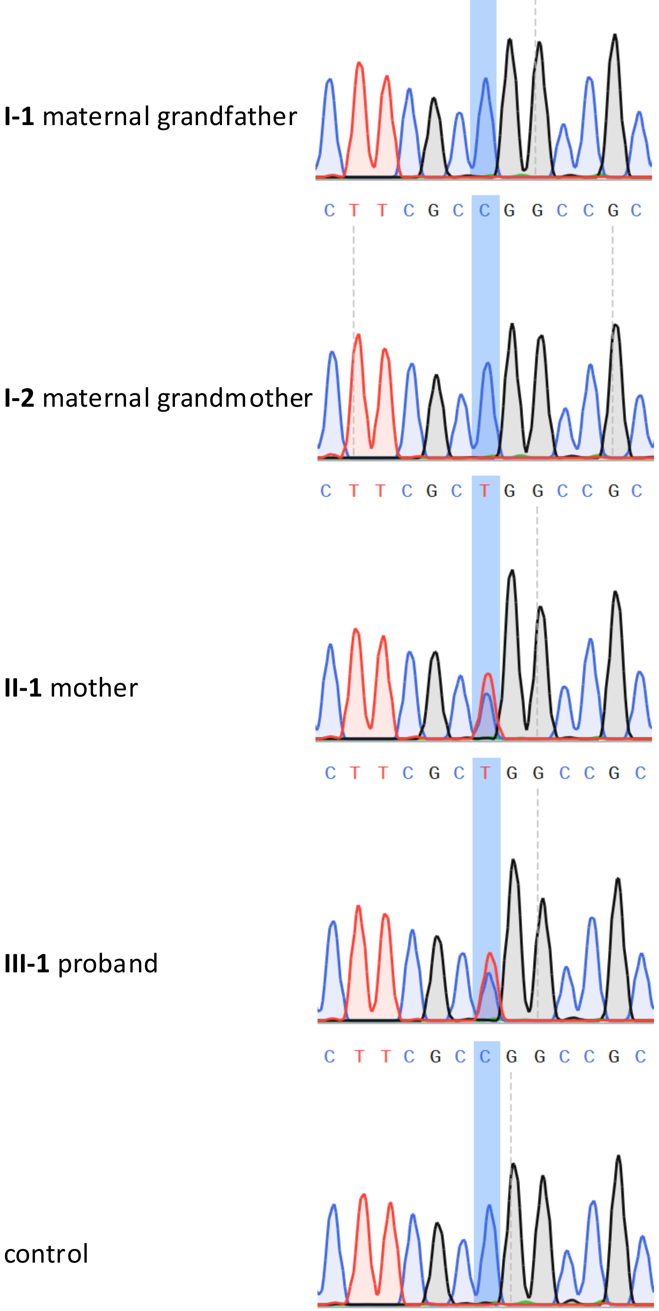

HGVS (NC_000003.12): g.147413391C>T
NM_003412.4 :c.1184C>T

Description:
Family 20 (pedigree to left) were enrolled in the 'Genetics of Craniofacial Malformations' study. The Mother II-1 was more mildly affected (indicated by grey shading in the pedigree) than her daughter III-1 (see Supplemental Table 3). The grandparents were unaffected.

Methods:
DNA was extracted from blood from all the family members shown in the pedigree, except from I-2 where DNA was extracted from saliva as described previously (Suppl. Fig. 1). Primers were designed for chr3:147,413,391-147,413,391. The following primers were used for amplification:

F = **acactctttccctacacgacgctcttccgatct**CACTGGCTCTTTATGTCCGTAAAA

R = **gtgactggagttcagacgtgtgctcttccgatct**ACGTGTGTGATCAGTCTCTTAAATAG

Highlighted in bold are TruSeq adapter sequences (R1+ R2)

PCR was carried out using Q5 HF Polymerase (NEB) followed by Sanger sequencing with the forward primer. The maternal grandparents were homozygous for the reference allele.

Illumina paired-end sequencing was conducted to generate deep next-generation sequencing data. PCR I was set up with Q5 HF Polymerase (NEB) to create amplicons with stubby ends to add the full Illumina adapters (P5 and P7 which contains the indexes) via a second PCR. After confirmation of the correct product size, PCR I product was barcoded with Illumina adapters using iProof (Bio-rad). The product was cleaned using AMPure beads in a ratio of 1:1. Pooled samples were sequenced by Novogene via the NovaSeq platform. Data analysis was carried out as described previously for Family 3, confirming a a heterozygous *de novo* variant in the mother (II-1) with no evidence of mosaicism (49% T allele).

Supplemental Figure 2. ZIC1 transactivation assays (individual replicates with representative western blot) & localisation

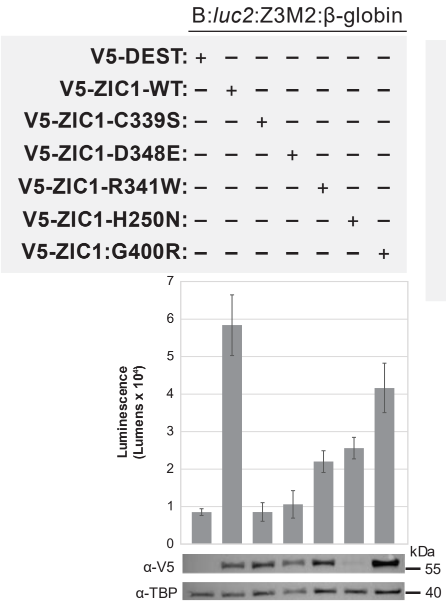

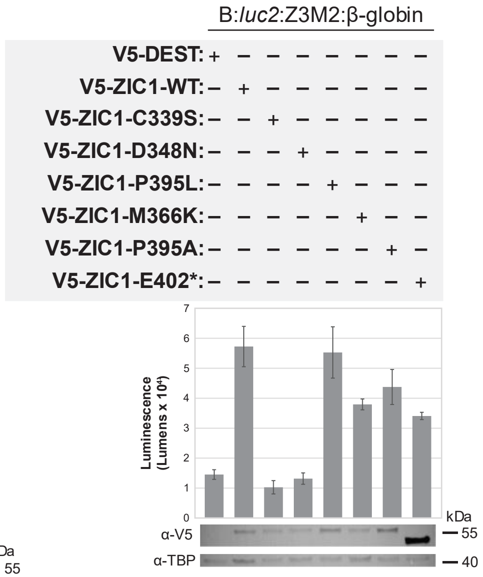

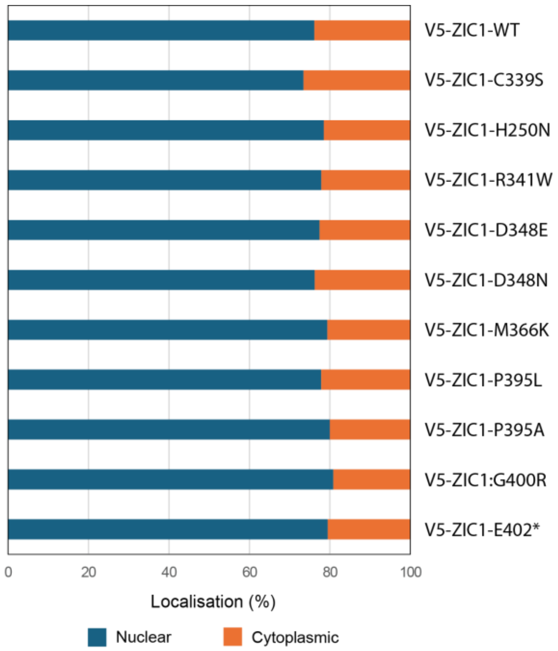

ZIC1 transactivation assay and Western blot.
In each transfection luminescence was measured 24 hours post-transfection in each of three replicate samples and each transfection repeated three times. The graphs show one representative experiment with the corresponding western blot. Error bars represent SD between the three internal replicates. Expression was confirmed with α-V5, and the α-TBP blot served as nuclear fraction loading control.

Quantification of the subcellular distribution of ZIC1 variants.
Subcellular localisation of transfection V5-ZIC1 protein was quantified using Harmony™ 5.2 software. Blue and orange regions represent the nuclear and cytoplasmic compartments respectively.

Methods:

Transactivation & Westerns: Raw luminescence data from three independent biological repeats were pooled for each experiment, and a one-way ANOVA was performed using R 4.5.0, with technical replicates included as a covariate. The mean and standard deviation for each transfection was calculated for all treatment groups. The mean relative luminescence (RLA) value, calculated by normalising the mean luminescence value of each treatment group to V5-DEST such that the V5-DEST value is equal to 1, was determined from three experimental repeats. Normalised standard error of the mean (SEM) for RLA was calculated by dividing the SEM of raw luminescence values by the mean luminescence value of V5-DEST from three independent repeats. A post hoc test was performed using the Tukey's Honestly Significant Difference (HSD) method ($\alpha = 0.01$) to identify treatment groups that were significantly different. Treatment groups were significantly different if the mean difference between two groups was larger than the HSD value. The different letters (a, b, c…) denote statistical differences, where treatment groups with the same letter are not statistically significant. Analysis of variance showed a large effect size, $F(11, 123) = 50.406$, $p = 2.89e-40$, $\eta 2 = 2.12$.

Subcellular locsalisation: V5-tagged ZIC1 expression constructs containing the relevant mutations were transfected into HEK293T cells. Each transfection was plated in triplicate onto a 96-well black, clear bottom plate (PhenoPlate, 6055300) coated with 0.05 mg/mL of poly-D-lysine (Sigma-Aldrich) for 2h. 24h post-transfection, cells were fixed with 4% paraformaldehyde (PFA; Electron Microscopy Sciences), permeabilised with 0.25% Triton X-100 (Sigma-Aldrich) and blocked in 5% skim milk powder (5% w/v Diploma brand skim milk powder in 1X PBS) for 1h at room temperature. Cells were incubated with primary antibody α-V5 (1:200 dilution; Abcam, ab9116) in blocking solution for 1h at room temperature, followed by incubation with secondary antibody α-rabbit Alexa Fluor 488 (1:500 dilution; Invitrogen A21206) for 1h at room temperature. 2 μg/mL of DAPI was then added for 10 min at room temperature. To detect the plasma membranes, cells were stained with CellMask™ Deep Red Plasma Membrane Marker (1 μg/mL, Invitrogen, C10046) for 20 min at room temperature before imaging with the Revvity Opera Phenix High-Content Imagine Microscope. Subcellular localisation of transfection V5-ZIC1 protein was quantified using Harmony™ 5.2 software.

Supplemental Figure 3. Sequence conservation of ZF4 and in silico structural analysis of p.Arg341Trp (NCBI:NP_003403.2)

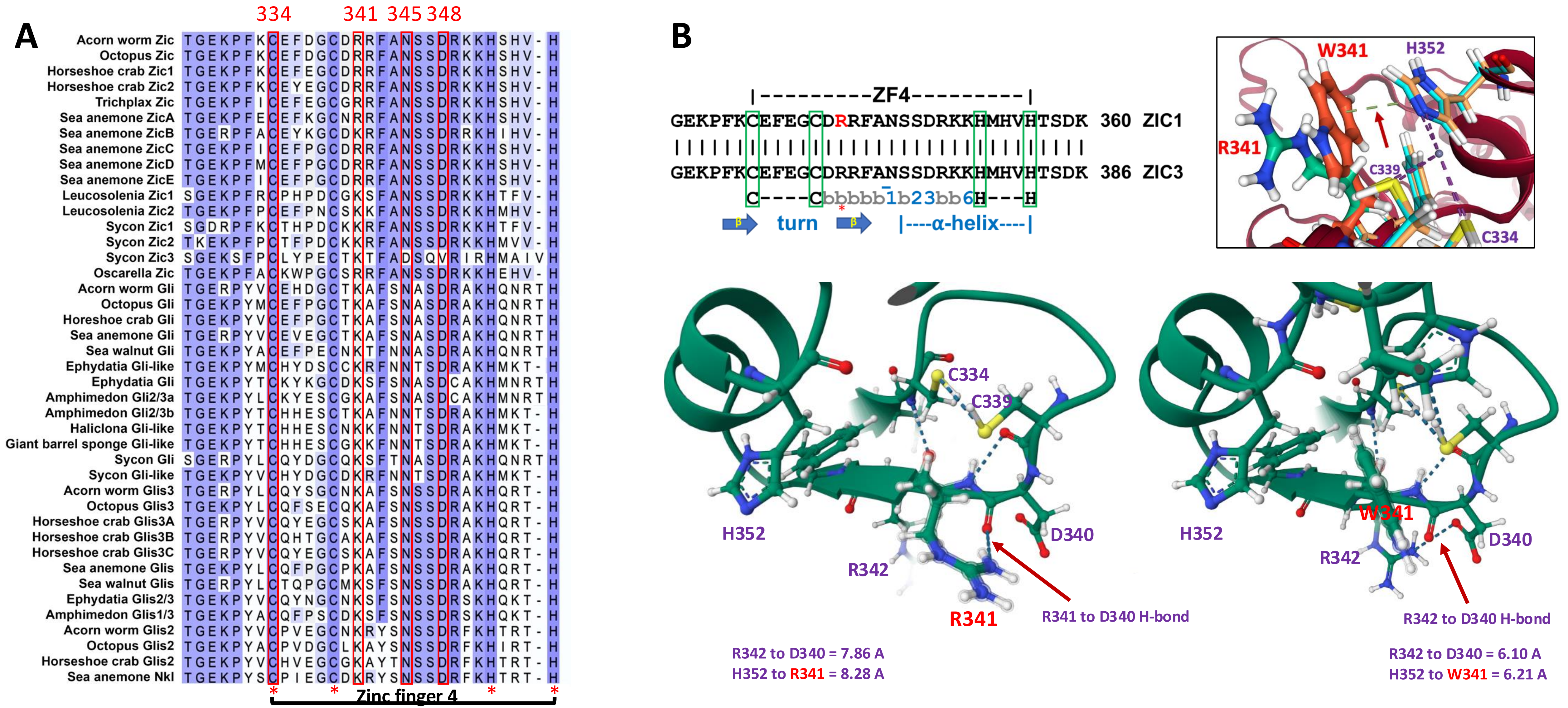

A. Amino acid sequence alignment of Zic/Gli/Glis paralogues from Bilateria (Deuterostomia and Protostomia), Porifera, Cnidaria, Ctenphora and Placazoa[29]. The position of the amino acids substituted in ZF4 are indicated by red boxes (p.Cys341Gly; p.Arg341Trp; p.Asn345Lys; p.Asp348Glu). The ZF C2H2 residues are indicated by asterisks. **B**. Top left, sequence of the ZIC1 zinc finger 4 motif aligned to ZIC3 (100% identity) showing key residues of C2H2 zinc fingers, and position of the two β sheets and α helix. Arginine 341 is in red lettering, the canonical cysteine and histidine residues are boxed in green, the DNA interacting residues are shown in blue, and the phosphate backbone interacting residues in grey. After the histidine at position 7, the second most conserved phosphate backbone contact is at position 1 of the second strand, i.e two residues along from the 2nd cysteine, and is most often a basic residue (red asterisk). Top right, overlay of 3D structures for R341 and W341 generated using MichalaNGLo – Venus (https://michelanglo.sgc.ox.ac.uk/), based on the ZIC3 structure (https://www.rcsb.org/structure/2rpc) (Tomizawa et al., 2009). Venus predicted a structurally neutral effect. The red arrow indicates a new potential interaction between the aromatic rings of Trp341 and His352. Bottom, comparison of 3D structures for R341 (left) and W341 (right) generated using MichalaNGLo – Venus, based on the ZIC3 structure and analysed using using MolStar (https://www.rcsb.org/3d-view). Arrows indicate the change in H-bonding from Asp340 to Arg342 (rather than Arg341) in the presence of Trp341. Distances between amino acids were calculated using MolStar and are shown below the structures. p.R341W substitution results in reduced distance between the indicated residues.

Supplemental Figure 4. Predicted binding specificity changes for ZIC1 zinc finger substitutions (NCBI:NP_003403.2)

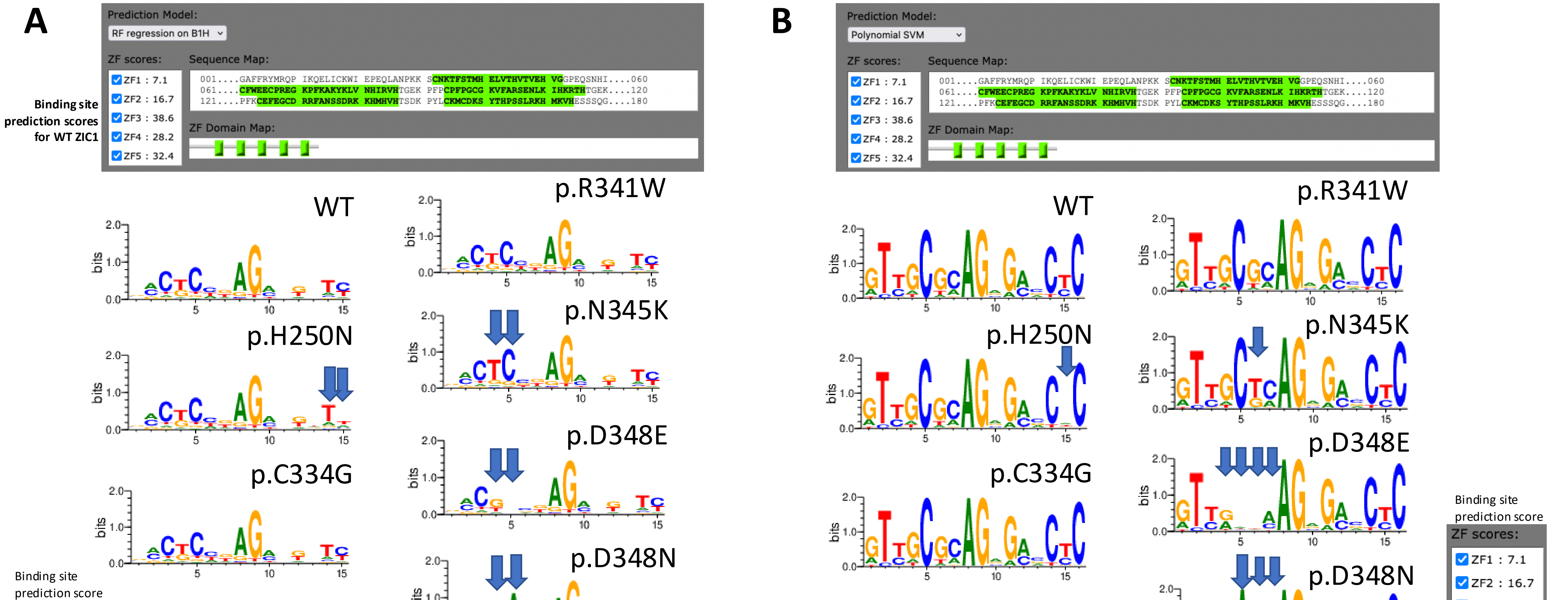

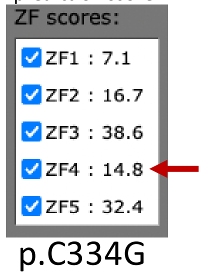

Potential DNA binding specificity changes were assessed using a web tool for predicting DNA recognition by C2H2 zinc finger proteins (http://zf.princeton.edu/index.php; Persikov et al., 2009; 2014). Two prediction models were used: **A**, Model based on a library of C2H2-ZF protein to DNA interfaces generated using a bacterial 1 hybrid (B1H) approach, and **B**, based on inferring statistical pairwise contact energies between ZF residues and the corresponding nucleotides in DNA via support vector machines (SVMs). Top boxes, sequence from ZIC1 used in the analyses. Each zinc finger is scored and highlighted. ZF1 has a low score and is not fully highlighted (the first cysteine is at amino acid number 3), presumably because it is an atypical C2H2 zinc finger motif with increased distance between the first two cysteines. Below, sequence logos predicted by the two models, with the amino acid substitution indicated above each logo. Arrows indicate any changes to the WT predicted sequence logo. The DNA contacting amino acids that are substituted are His250 (position +2), Asn345 (position -1) and Asp348 (position +3). Bottom left, p.C334G had a significant impact on the zinc finger score was (arrowed; most confident ZF motifs have a score of over 17.7, and with cysteine mutated the ZF score is 14.8). p.D348N increased the ZF4 binding score (bottom right).

Supplemental Figure 5. p.His250Asn in silico structural analysis and conservation

(NCBI:NP_003403.2)

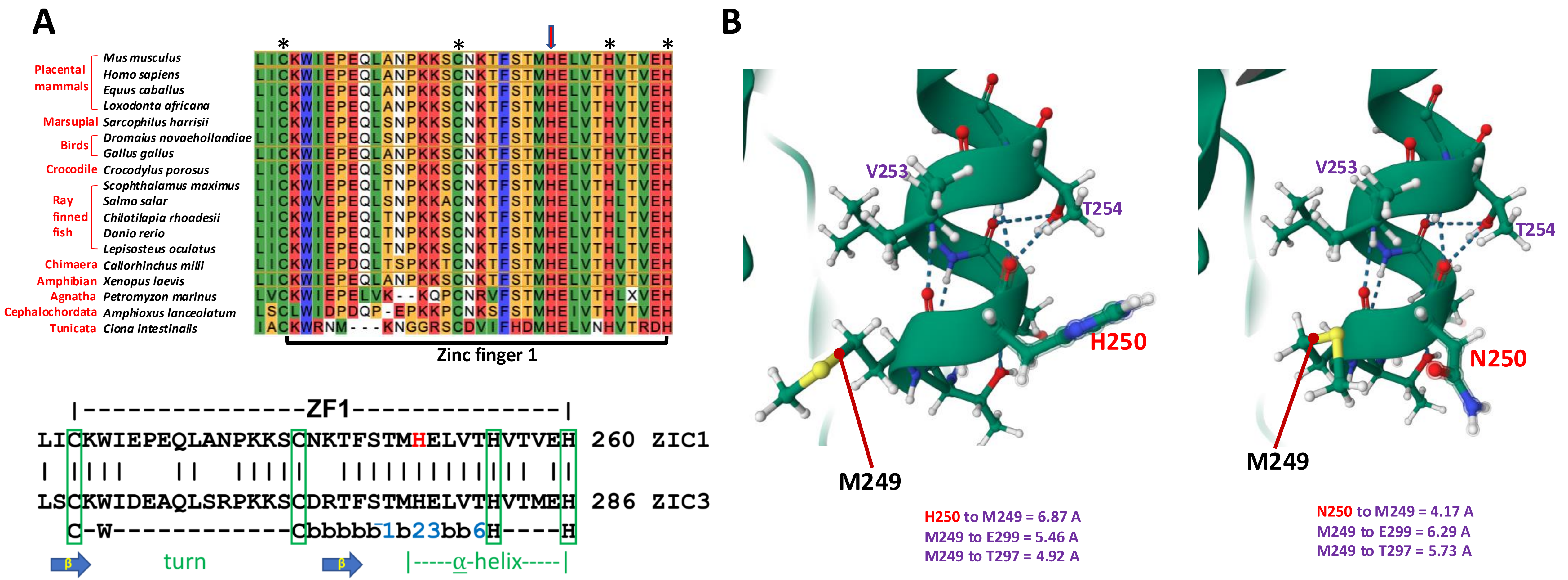

A. Top, amino acid sequence alignment of Zic1 proteins from Chordates. The position of the Zic1 His250 in ZF1 is indicated by the arrow. The cysteines, and histidines of zinc finger C2H2 motif are indicated by asterisks. The amino acids sequences were retrieved and aligned in Uniprot (https://www.uniprot.org/align). Bottom, sequence of the ZIC1 zinc finger 4 motif aligned to ZIC3 (76% identity) showing key residues of C2H2 zinc fingers, and position of the two β sheets and α helix. Histidine 250 is in red lettering, the canonical cysteine and histidine residues are boxed in green, the DNA interacting residues are shown in blue, and the phosphate backbone interacting residues in grey. Histidine 250 is at the +2 position of the zinc finger.

B. comparison of 3D structures for H250 (left) and N250 (right) generated using MichalaNGLo – Venus (https://michelanglo.sgc.ox.ac.uk/) and based on the ZIC3 structure (https://www.rcsb.org/structure/2rpc)(Tomizawa et al., 2009). Venus predicted a structurally neutral effect. The adjacent amino acid M249 is highlighted; the presence of N250 leads to an alteration in orientation of this residue, changing the distance between the α helices of ZF1 and ZF2 which are packed against each other via hydrophobic interactions (Hatayama et al., 2008). Distances between amino acids were calculated using MolStar (https://www.rcsb.org/3d-view) and are indicated below the structures.

Supplemental Figure 6. In silico structural analysis of p.Asn345Lys (NCBI:NP_003403.2)

A

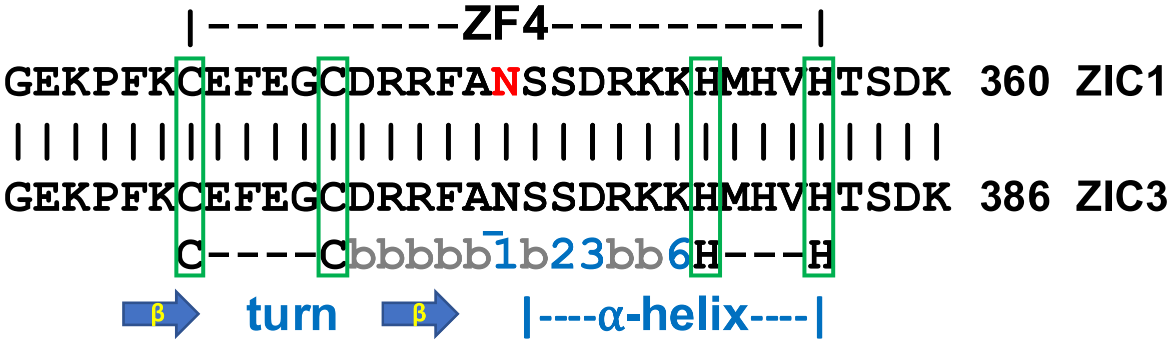

A. Sequence of the ZIC1 zinc finger 4 motif aligned to ZIC3 (100% identity) showing key residues of C2H2 zinc fingers, and position of the two β sheets and α helix. Asparagine 345 is in red lettering, the canonical cysteine and histidine residues are boxed in green, the DNA interacting residues are shown in blue, and the phosphate backbone interacting residues in grey. Asparagine 345 is at the -1 position of the zinc finger. **B**. Comparison of 3D structures for N345 (left) and K345 (right) generated using MichalaNGLo – Venus (https://michelanglo.sgc.ox.ac.uk/), based on the ZIC3 structure (https://www.rcsb.org/structure/2rpc)(Tomizawa et al., 2009) and analysed using using MolStar (https://www.rcsb.org/3d-view). Venus predicted a structurally neutral effect. Arrows indicate H-bonds that are lost in the predicted K345 configuration. Pro331 is a conserved residue within the linker region between ZF3 and ZF4. It is part of the TGEKP consensus sequence often found between adjacent ZFs that contact DNA, and has been shown to be important for high affinity DNA binding. In ZIC1 TGEKP is also found between ZF2 and ZF3. Distances between amino acids were calculated using MolStar and are indicated below the structures.

B

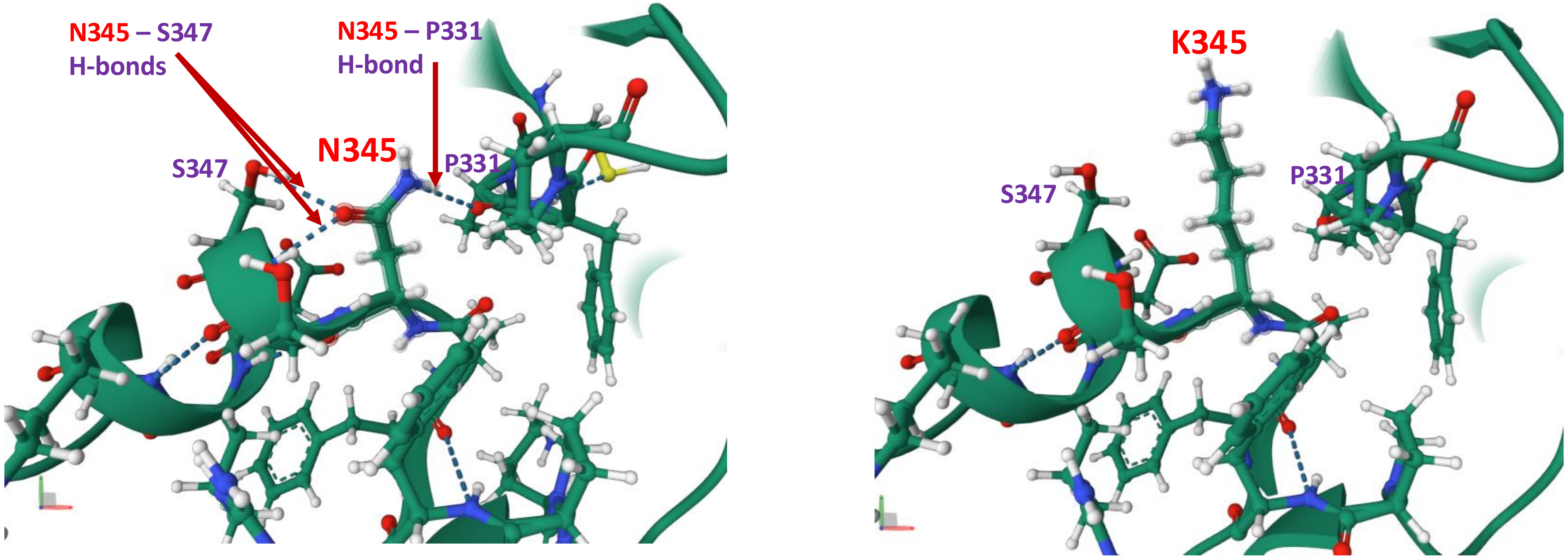

N345 to P331 = 5.97 A
N345 to S347 = 4.43 A

K345 to P331 = 6.64 A
K345 to S347 = 4.32 A

Supplemental Figure 7. p.Asp348Glu in silico structural analysis and conservation (NCBI:NP_003403.2)

A

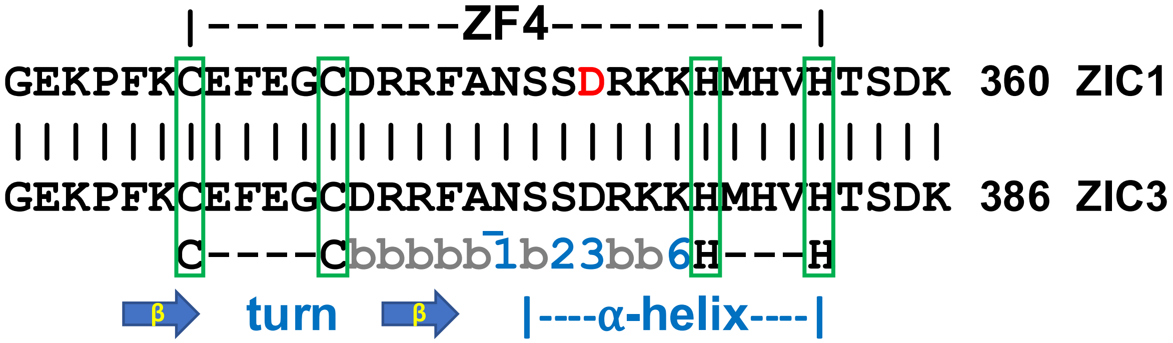

A. Sequence of the ZIC1 zinc finger 4 motif aligned to ZIC3 (100% identity) showing key residues of C2H2 zinc fingers, and position of the two β sheets and α helix. Aspartate 348 is in red lettering, the canonical cysteine and histidine residues are boxed in green, the DNA interacting residues are shown in blue, and the phosphate backbone interacting residues in grey. Asparagine 348 is at the +3 position of the zinc finger. **B**. Comparison of 3D structures for D348 (left), E348 (middle) and N348 (right) generated using MichalaNGLo – Venus (https://michelanglo.sgc.ox.ac.uk/) and based on the ZIC3 structure (https://www.rcsb.org/structure/2rpc)(Tomizawa et al., 2009). Venus predicted a structurally neutral effect. Arrows on the left indicate H-bonds from D348, including to N345 at the -1 position, that are maintained in E348 structure. A red arrow indicates a potential new H-bond between E348 and K351 (absent in D348). Lysine 351 is at position +6. New H-bonds in the N348 structure are visible between N348 and canonical histidine of ZF4, H352.

B

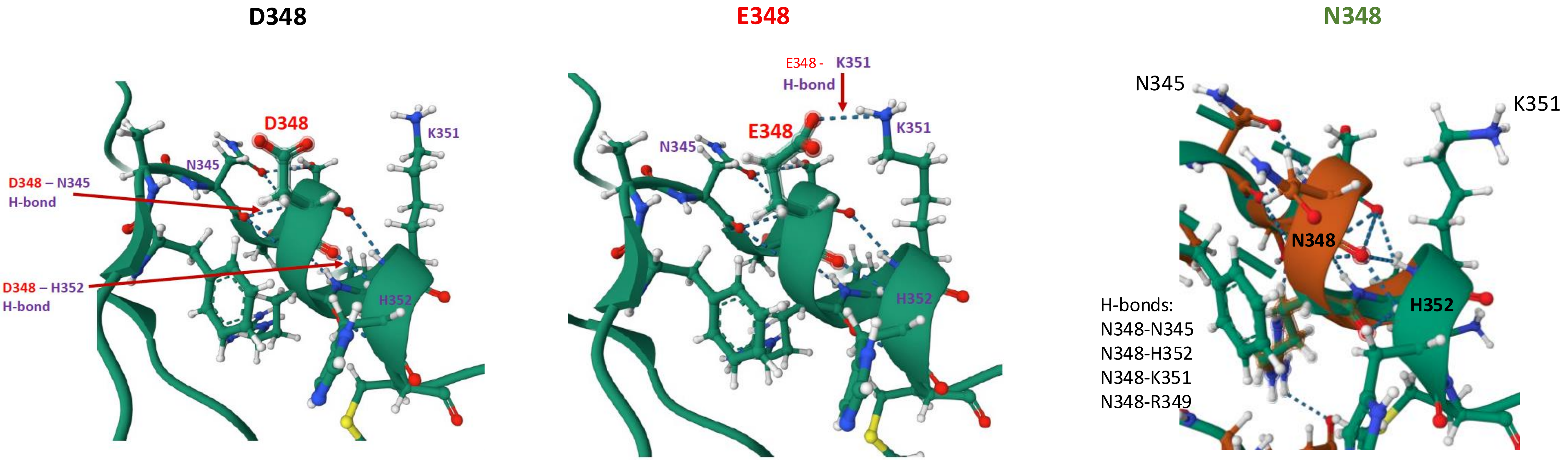

Supplemental Figure 8. p.Met366Lys in silico structural analysis and conservation (NCBI:NP_003403.2)

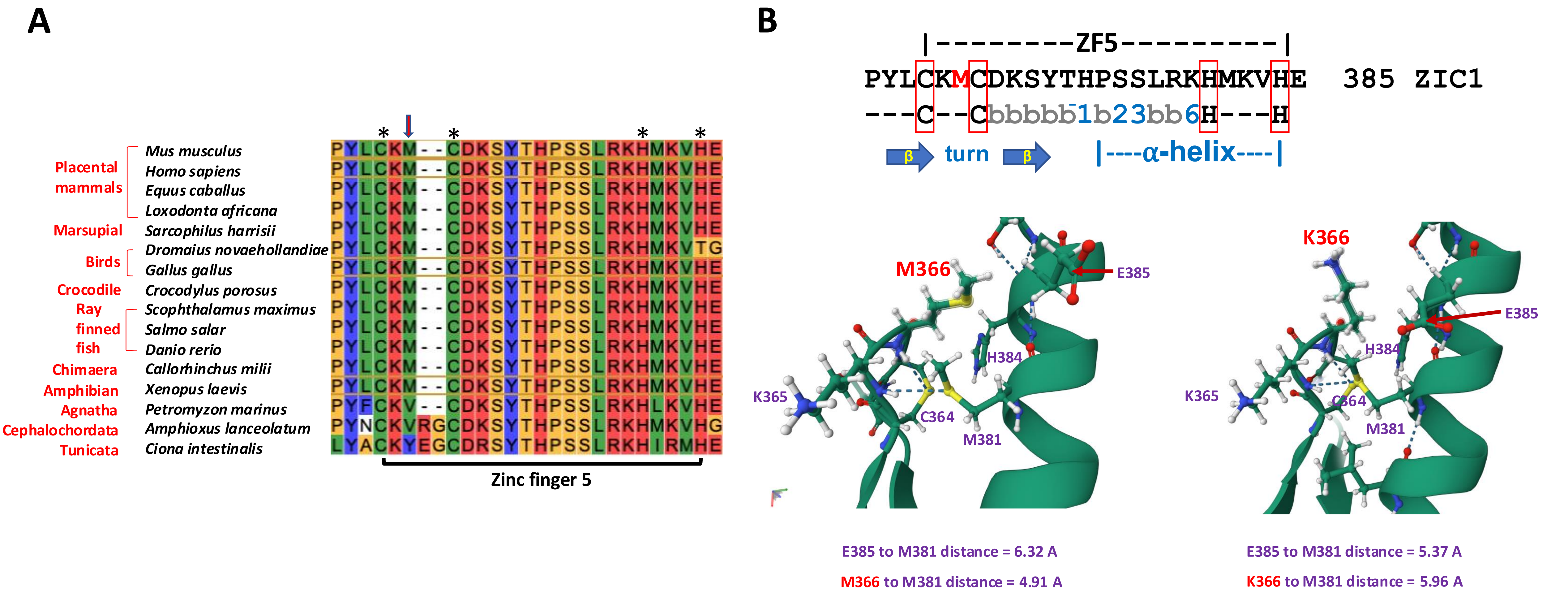

A. Amino acid sequence alignment of Zic1 proteins from Chordates. The position of the Zic1 Met366 in ZF5 is indicated by the arrow. The cysteines, and histidines of zinc finger C2H2 motif are indicated by asterisks. The amino acids sequences were retrieved and aligned in Uniprot (https://www.uniprot.org/align). **B**. Top, sequence of the ZIC1 zinc finger 5 motif showing key residues of C2H2 zinc fingers, and position of the two β sheets and α helix. The canonical cysteine and histidine residues are boxed in red, the DNA interacting residues are shown in blue, and the phosphate backbone interacting residues in grey. Bottom, comparison of 3D structures for M366 (left) and K366 (right) generated using MichalaNGLo – Venus (https://michelanglo.sgc.ox.ac.uk/) and RCSB PDB MolStar (https://www.rcsb.org/3d-view), based on an AlphaFold structure prediction (https://alphafold.ebi.ac.uk/entry/Q15915) as no other structural data is available. Distances between amino acids were calculated using MolStar and are indicated below the structures. Venus predicted a structurally neutral effect. The red arrow in the K366 structure prediction indicates an apparent different orientation of the Glu385 residue.
